# Targeting AKT with costunolide suppresses the growth of colorectal cancer cells and induces apoptosis in vitro and in vivo

**DOI:** 10.1186/s13046-021-01895-w

**Published:** 2021-03-30

**Authors:** Hai Huang, Song Park, Haibo Zhang, Sijun Park, Wookbong Kwon, Enugyung Kim, Xiujuan Zhang, Soyoung Jang, Duhak Yoon, Seong-Kyoon Choi, Jun-koo Yi, Sung-hyun Kim, Zigang Dong, Mee-hyun Lee, Zaeyoung Ryoo, Myoung Ok Kim

**Affiliations:** 1 Department of Animal Science and Biotechnology, ITRD, Kyungpook National University, Sangju, 37224 Republic of Korea; 2 Core Protein Resources Center, DGIST, Daegu, Republic of Korea; 3 Department of Brain and Cognitive Sciences, DGIST, Daegu, Republic of Korea; 4 School of Life Science, Kyungpook National University, Daegu, Republic of Korea; 5 Division of Biotechnology, DGIST, Daegu, Republic of Korea; 6 Department of Pulmonary and Critical Care Medicine, Huashan Hospital, Fudan University, Shanghai, 200040 China; 7 Gyeongsangbuk-do Livestock Research Institute, Yeongju, South Korea; 8 Department of Bio-Medical Analysis, Korea Polytechnic College, Chungnam, Korea; 9 China-US (Henan) Hormel Cancer Institute, Zhengzhou, 450008 Henan China; 10 College of Korean Medicine, Dongshin University, Naju, Jeollanamdo 58245 Republic of Korea

**Keywords:** Colon cancer, Costunolide (CTD), AKT, AKT/MDM2/p53 pathway, Ubiquitination, Xenograft model

## Abstract

**Background:**

Colorectal cancer (CRC) is a clinically challenging malignant tumor worldwide. As a natural product and sesquiterpene lactone, Costunolide (CTD) has been reported to possess anticancer activities. However, the regulation mechanism and precise target of this substance remain undiscovered in CRC. In this study, we found that CTD inhibited CRC cell proliferation in vitro and in vivo by targeting AKT.

**Methods:**

Effects of CTD on colon cancer cell growth in vitro were evaluated in cell proliferation assays, migration and invasion, propidium iodide, and annexin V-staining analyses. Targets of CTD were identified utilizing phosphoprotein-specific antibody array; Costunolide-sepharose conjugated bead pull-down analysis and knockdown techniques. We investigated the underlying mechanisms of CTD by ubiquitination, immunofluorescence staining, and western blot assays. Cell-derived tumour xenografts (CDX) in nude mice and immunohistochemistry were used to assess anti-tumour effects of CTD in vivo.

**Results:**

CTD suppressed the proliferation, anchorage-independent colony growth and epithelial-mesenchymal transformation (EMT) of CRC cells including HCT-15, HCT-116 and DLD1. Besides, the CTD also triggered cell apoptosis and cell cycle arrest at the G2/M phase. The CTD activates and induces p53 stability by inhibiting MDM2 ubiquitination via the suppression of AKT’s phosphorylation in vitro. The CTD suppresses cell growth in a p53-independent fashion manner; p53 activation may contribute to the anticancer activity of CTD via target AKT. Finally, the CTD decreased the volume of CDX tumors without of the body weight loss and reduced the expression of AKT-MDM2-p53 signaling pathway in xenograft tumors.

**Conclusions:**

Our project has uncovered the mechanism underlying the biological activity of CTD in colon cancer and confirmed the AKT is a directly target of CTD. All of which These results revealed that CTD might be a new AKT inhibitor in colon cancer treatment, and CTD is worthy of further exploration in preclinical and clinical trials.

**Supplementary Information:**

The online version contains supplementary material available at 10.1186/s13046-021-01895-w.

## Background

Globally, colorectal cancer (CRC) is common and the third-most leading cause of cancer mortality in humans [1]. Currently, fluorouracil and oxaliplatin or their combination remain as the first choice drug against CRC. Furthermore, combining oxaliplatin with FU or capecitabine has proven beneficial in clinical trials [2]. However, it remains uncertain whether all patients with CRC reap adjuvant chemotherapy benefits to testify its related toxicity, inconvenience, and expense. The development of targeted therapies against CRC has not progressed as rapidly or smoothly as expected over the past few decades. Previously, drugs targeting VEGF, and EGFR have been used in clinical trials. Such medications should be further examined. Therefore, the ongoing cancer agents’ research is vital in developing novel approaches to prevent, treat, and improve the survival rates of patients with CRC.

The AKT is a serine/threonine kinase, also known as Protein Kinase B (PKB), that inhibits apoptosis, regulates glycogen metabolism, and contributes toward cancer progression [3, 4]. Overexpression of phosphorylated AKT is a therapeutic target for the treating of malignant tumors. Previous studies have showed that the AKT-MDM2-p53 signaling pathway has a considerable effect on cell apoptosis and is related to the development and progression of many cancers, including colorectal cancer [5–8]. It has been reported that AKT-mediated MDM2 phosphorylation induces apoptosis through activation of p73 and E2F1 in p53 deficient colon cancer cell [9]. However, the mechanism of the AKT-MDM2-p53 pathway in CRC cells has not been explained in detail. Furthermore, p-AKT is shown to indirectly regulate p53, which acts as a crucial tumor suppressor in multicellular organisms via different mechanisms, such as the MDM2 [10–12]. The MDM2 acts as a bridge between AKT and p53 and is an excellent substrate of AKT. Therefore, inhibiting the MDM2 phosphorylation could promote the p53 function in cancer therapies. Besides, p53 activation may downregulate the AKT/mTOR pathway via an AMP kinase (AMPK)-mediated mechanism [13] or by PTEN upregulating [14], resulting in anticancer activity. Conversely, MDM2 can also be regulated by p53-induced negative feedback via AKT inhibition [15]. Thus, the AKT-MDM2-p53 pathway plays an essential role in cancers and targeting AKT may be an ideal approach for anticancer drug development.

The p53 is a crucial tumor suppressor protein that is mutated in more than half of all types of human cancers. Moreover, it is closely associated with cellular stresses and responses such as DNA damage, apoptosis, and senescence [16, 17]. Focus on the protein–protein interactions of p53 as a proof of concept for cancer therapies [18–20] have allowed many studies to determine the therapeutic use of p53 activators, which is no longer a dream. FAM3B, SPARC, LACTB, and PIG3 are involved in regulating the balance between p53 and MDM2 on the AKT-MDM2-p53 signaling pathway in various cancers, including colon cancer [21–24]. Therefore, the p53-MDM2 protein interaction inhibitors are examined and applied as anticancer drugs [25]. Thus, interfering or suppressing the p53–MDM2 protein interaction is an effective way of preventing or treating cancer in the future. These studies suggest that the balance between the oncogene and the suppressor gene is essential for cancer therapy. In this study, we examined the effects of natural compounds on MDM2-p53 by targeting AKT.

Several natural products mediate anticancer activities by targeting oncogenes or activating tumor suppressors. Hence, in this study, we introduce costunolide, which exhibits a potential anticancer action by suppressing proliferation and inducing apoptosis in a broad range of human cancers in vitro *and* in vivo*,* such as colon cancer [26–29]. A study [30] showed that the potential targets of CTD are intracellular kinases (MAP kinases), transcription factors (NF-κB, AP-1), and expression of proinflammatory mediators (COX-2, iNOS). However, a direct molecular target for CTD is yet to be identified. Therefore, this prompts us to search for a directly molecular target of CTD to exert its anticancer effects. The objective of this study was to identify a novel AKT inhibitor and to investigate the efficacy of the newly discovered AKT inhibitor, CTD, in the prevention or treatment of colon cancer. This study also provides a reliable fundamental for the preclinical study of CTD.

## Methods

### Reagents

Costunolide (CTD, purity ≥98%, CAS: 553–21-9, Fig. 1a) was purchased from Chengdu Biopurify Phytochemicals Co. Ltd. (China) and validated it by high- performance liquid chromatography. MTT (3-[4,5-dimethylthiazol-2-yl]-2,5-diphenyl -tetrazoliumbromide, Ruitaibio, KOREA), the antibodies against phosphorylated AKT (ser473), total AKT, phosphorylated MDM2 (Ser166), phosphorylated p53 (ser315), and total p53 were purchased from Cell Signaling Technology (Beverly, MA, USA total MDM2, phosphorylated CDC25C, total CDC25C, cyclin B1, Bcl-2, and β-actin from Santa Cruz Biotechnology (Santa, Cruz, CA, USA).

**Fig. 1:**
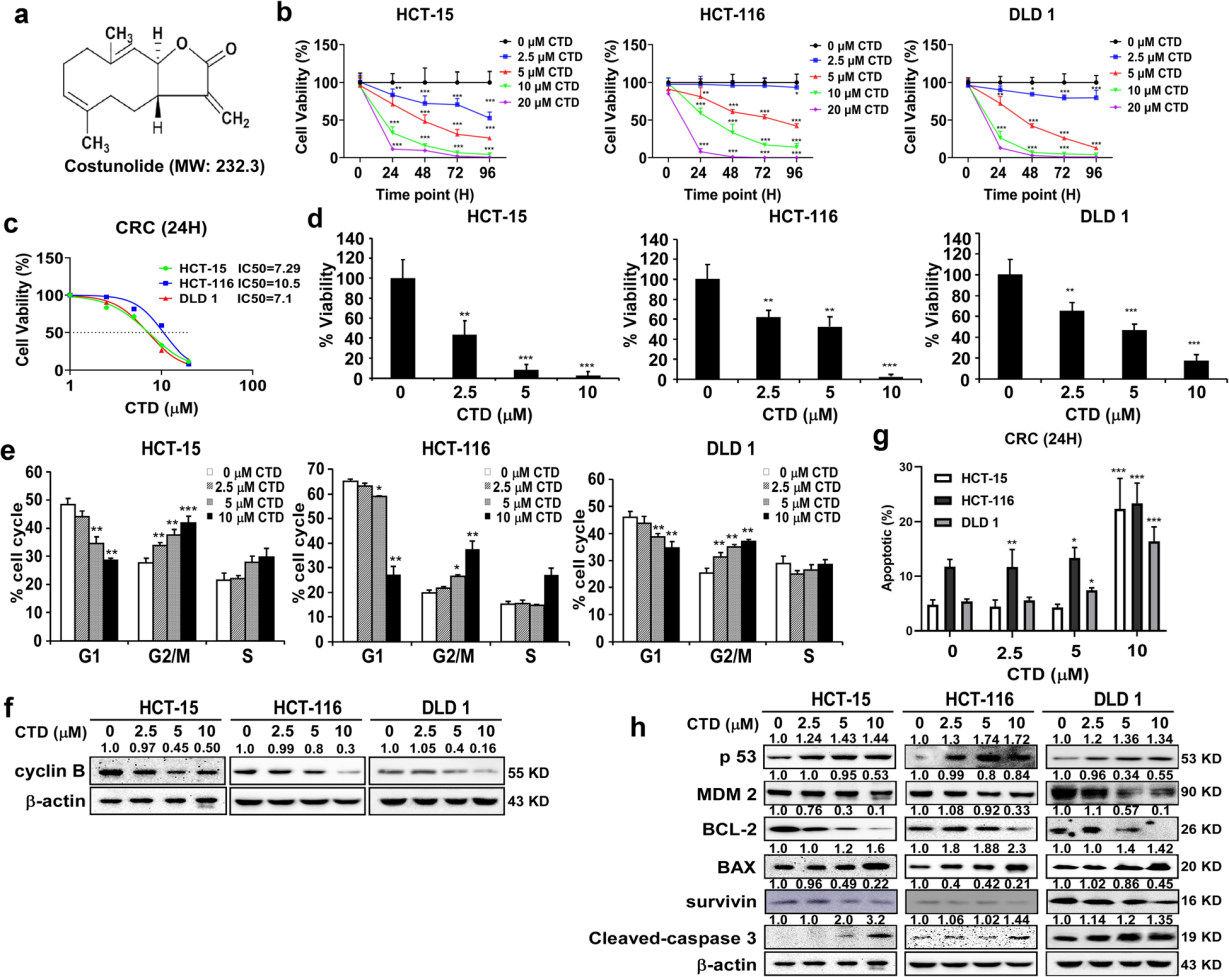
CTD inhibits the proliferation of the CRC cells. **a** The structure of the costunolide. **b** MTT assay was conducted to measure the effect of CTD with various concentrations on the proliferation of the CRC cells at 24, 48, 72, and 96 h, respectively. **c** IC_50_ was analyzed via the cell viability assay. **d** The anchorage-independent colony formation assay was performed to detect the proliferative ability of the CRC cells treated with different concentrations of CTD for 2 weeks. **e** The cell cycle of the CRC cells treated with different concentrations of CTD for 24 h was detected using flow cytometry. **f** The effects of CTD on the expression of the biomarkers with the cell cycle. **g** The cell apoptosis of the treated with various CTD concentrations for 24 h was detected using flow cytometry. **h** The effects of CTD on the expression of biomarkers of apoptosis. The data are shown as mean ± SD of values from triplicate samples. (*p < 0.05, **p < 0.01, ***p < 0.01) indicate a significant difference compared to the control

### Cell lines and culture

Human colorectal cancer cell lines (HCT-15, HCT-116, and DLD1) were purchased from the Korea Cell Lines Bank (KCLB). Professor Mee-Hyun Lee (Dongshin University, KOREA) kindly provided us with the HCT-116 (p53+/+ and p53−/−) cell lines. The human immortalized healthy colon cell CCD-18Co (cultivate in MEM) from KCLB. Cells were cultivated in the following medium (obtained from Gibco by Life Technologies): MEM (CCD-18Co), RPMI 1640 (HCT-15 and DLD1), and McCoy’s 5A (HCT-116 and HCT-116 p53+/+ or p53−/−) supplemented with penicillin–streptomycin (1%) (Solarbio Technology Co., Ltd. Korea) and 10% fetal bovine serum (Biological Industries) in a humidified atmosphere at 37 °C with 5% CO_2_. All the cells were cytogenetically tested and authenticated before being frozen. Each vial of frozen cells was thawed and maintained in culture for a maximum of 8 passages.

### Cell viability assay

CRC cells (2 × 10^4^ cells) and healthy colon cells (5 × 10^4^ cells) were seeded into 96-well plates and treated them with different doses of CTD or dimethyl sulfoxide (DMSO, ≥99.7%, Sigma-Aldrich Co. LLC). Cell proliferation was determined using the MTT reagent every 24 h for 4 consecutive days. Absorbance was measured at 570/690 nm. For anchorage-independent cell growth assessment, the cells (8 × 10^3^ cells/well) suspended in complete medium were added to 0.3% agar with CTD (0, 2.5, 5, and 10 μM) for anchorage-independent cell growth assessment in the top layer, over a base layer of 0.6% agar with CTD. The cultures were maintained at 37 °C in a 5% CO_2_ incubator for 2 weeks, and the colonies were then visualized under a microscope and counted using the Image-Pro Plus software (v.6.1) (Media Cybernetics).

### Flow cytometry analyses

Cells (2 × 10^5^) were treated with CTD or DMSO in 60-mm dishes and incubated for 24 h before analysis. The cells were trypsinized, washed with PBS, and fixed in 70% ethanol at − 20 °C overnight, followed by incubation with RNase (1 mg/mL) and staining with propidium iodide (PI) (50 μg/mL). DNA content was determined via flow cytometry using a BD FACS Calibur instrument. Cell death was quantified using the AnnexinV Alexa Fluor 488 & Propidium Iodide (PI) Dead Cell Apoptosis kit from Invitrogen. In brief, cells were harvested and analyzed with a BD FACS Calibur instrument according to the manufacturer’s instructions. The cells that were positive for Annexin V alone and for Annexin V and PI were counted.

### Transwell migration, invasion assay, and wound-healing assay

Transwell chambers (8-μm pore size, COSTAR) were used to conduct the migration and invasion assays as per the manufacturer’s procedure and published methods [31]. For the cell transmembrane migration assay, all processes were performed similarly to those in the invasion assay except for matrigel (1:5) coating. In brief, cells (1 × 10^5^) were treated with CTD or DMSO in chambers for 24 h. Cells were fixed in 4% formaldehyde, permeabilized with 100% methanol, and stained with 0.5% crystal violet. The cells were photographed using a Leica SP2 confocal microscope (Leica Microsystems, Exton, PA) and then quantified them using the Image-Pro Plus software (v.6.1) (Media Cybernetics). For the scratch wound-healing assay, cells were seeded into 6-well plates for 24 h. A straight scratch across the cell layer with a sterile pipette tip, washed it with PBS, and then treated it with CTD for 24 h. The cells were photographed and the area was calculated using the Image-Pro Plus software (v.6.1).

### Phosphoprotein-specific antibody array

Cancer signaling phospho-antibody microarray (PCS248) was conducted using HCT-15 cells with 248 antibodies (Full Moon Biosystems, Inc.). Each antibody had two copies on coated glass microscope slides, and both positive and negative controls were established. The experiment of the antibody array was performed following the established protocol by Wayen Biotechnology (Deagu, Korea). The fluorescence signal of each antibody was obtained by the fluorescence intensity of the antibody spots. The protein phosphorylation degree was determined by ratio calculation as follows: phosphorylation ratio = (phosphoprotein/total protein). Validation of the microarray result was performed via western blotting.

### Western blot assay

Cells were lysed by lysis buffer (150 mmol/L NaCl, 1% NP-40, 50 mM Tris–HCl with 1 mM PMSF and protease inhibitor, and dephosphorylate inhibitor tablets mixture) for total protein collection. As per the procedure of the Nuclear-Cytosol Extraction Kit (Key GEN Technologies Inc., Jiangsu, China) to separate the nuclear and cytoplasmic proteins from total cellular proteins. The protein concentrations of the extracts of each group were quantified using the BCA protein kit (Thermo, USA). A same amount of protein was fractionated via SDS-PAGE and transferred onto polyvinylidene fluoride membranes (Millipore, Billerica, MA). Membranes were blocked using 5% non-fat dry milk in 1× TBST for 1 h and then incubated with primary antibodies overnight at 4 °C. Then, the membrane was washed with 1× TBST and incubated with the corresponding secondary antibody. Protein bands were visualized using the chemiluminescence reagent (GE Healthcare Life Science) with Da Vinci software.

### RNA extraction and qRT-polymerase chain reaction (PCR) analysis

Total RNA was extracted from the cells using the TRIzol® reagent (Invitrogen) as per the manufacturer’s instructions and was reverse-transcribed into cDNA by a reverse transcription kit (TAKARA, Tokyo, Japan). PCR amplification was conducted using the SYBR® Premix Ex Taq™ (Takara) on a Real-Time PCR Detection System. The results were normalized with β-actin, and each sample was measured in triplicate. Gene expression was calculated following the 2^-∆∆Ct^ method. Primer: p21 (F: TTCCGCACAGGAGCAAAGT; R: CGTCTCCGTGACGAAGTCAA).

### Pull-down assay using CNBr–costunolide-conjugated beads

CTD-Sepharose 4B beads were prepared following the manufacturer’s instructions (Amersham Pharmacia Biotech, GE Healthcare Bio-Science, Uppsala, Sweden). Cell lysates (500 μg) were incubated with CTD-Sepharose 4B beads or Sepharose 4B beads only in 1× lysis buffer (50 mM Tris–HCl pH 7.5, 5 mM EDTA, 150 mM NaCl, 1 mM dithiothreitol, 0.01% NP-40, 2 mg/ml bovine serum albumin, 20× protease inhibitor [1 tablet each]) at 4 °C with overnight rotation. After incubation, the beads were washed with a buffer (50 mM Tris–HCl pH 7.5, 5 mM EDTA, 150 mM NaCl, 1 mM dithiothreitol, 0.01% NP-40, and 0.2 mM PMSF). The AKT proteins bound to the beads were analyzed via immunoblotting.

### Immunoprecipitation (IP) assay

For the IP assay, cells were treated with CTD after seeding for 24 h, and the cell lysates (500 μg) were harvested and lysed in NP-40 lysis buffer (50 mM pH 7.4 Tris–HCl, 150 mM NaCl, 1% NP-40, 1 mM EDTA) containing the protease inhibitor phenyl methane sulfonyl fluoride (PMSF; 1:100) and protease inhibitor cocktail (Sigma). The lysates were incubated with primary antibodies and Dynabeads protein G (Sigma) at 4 °C with rotation overnight. The samples were washed with IP lysis buffer and the relevant protein binding was examined via immunoblotting.

### Detection of p53 ubiquitination in CRC cells via CTD treatment

Cells were seeded into a 10-cm dish and treated with cycloheximide (CHX, 10 μM) or MG132 (10 μM) at an indicated time point to examine protein stability. The samples were analyzed for p53 degradation via western blotting after harvesting. To assess ubiquitination, cells were transfected with pCDNA-flag-Ub. After 24 h of transfection and CTD treatment, the indicated dishes were treated with 10 μM CHX and MG132 for 2 h before harvest. The cells were lysed with a lysis buffer, and protein extracts (500 μg) were incubated with anti-flag antibody (M2) and rocked overnight at 4 °C. Then, 30 μl of Dynabeads Protein G was added to each tube and rocked for 4 h. The beads were collected by a magnet and washed with washing buffer and finally re-suspended in 50 μl of 1× protein sample buffer, followed by heating at 95 °C for 5 min. Ubiquitination was analyzed via immunoblotting.

### Lentivirus packaging and transfection

To establish stable cells for AKT knockdown, the lentivirus plasmids (shAKT1 and shAKT2) and packaging vectors (psPAX2 and PMD2.G) were co-transfected into HEK293T cells using transfection reagents (FuGENE HD, Promega, USA). Using a 0.45-μm filter, the viral particles were harvested via filtration and stored at − 80 °C. The cultured CRC cells were infected with virus particles in 8 μg/ml polybrene (Millipore, Billerica, MA) for 24 h. Then, cells were selected using puromycin (1 μg/ml) for 36 h, which were then used for subsequent experiments. For AKT overexpression, pUSE-CA-AKT1 and pUSE-CA-AKT2 plasmids were transfected into CRC cells using the 3-fold Transfection Reagent (FuGENE HD); the same amount of mock plasmid was transfected as a control. G418 (1 μg/ml) was used to select stable cells, which were then used for subsequent experiments. For rescue experiments, AKT1 and AKT2 genes were knocked down via lentiviral infection as described above for the HCT-116 cell line. Then, 1 mg/mL puromycin was utilized for selection. AKT1 or AKT2 was overexpressed in AKT knockdown cells by transfecting the pUSE-CA-AKT1/2 plasmid or mock plasmid directly and culturing for 12 h. Meanwhile, remaining dishes were maintained in 0.1 mg/mL puromycin for related experiments.

### Immunofluorescence assay

For immunofluorescence co-staining of p53 and MDM2, CRC cells were cultured on confocal dishes for 24 h and treated with CTD. The cells were washed with TBS and fixed in 4% paraformaldehyde for 15 mins, followed by permeabilization in 1% bovine serum albumin (BSA) containing 0.1% Triton X-100 and 1% BSA for blocking at 37 °C for 1 h. Later, incubated with antibodies against p53 (1:500) and MDM2 (1:500) with mild shaking at 4 °C overnight. Next, the cells were washed with PS and incubated with goat anti-rabbit (1:500) and goat anti-mouse 488 (1:500) secondary antibodies at room temperature for 3 h. Finally, nuclei were stained with 4′, 6-diamidino-2-phenylindole (DAPI) for 5 min. Cells were visualized with a Leica SP2 confocal microscope (Leica Microsystems, Exton, PA) and a photograph was taken.

### Xenograft model

The Kyung Pook National University Ethics Research Board (Dae-gu, South Korea) approved all animal research. Male athymic nude mice (5 ~ 6 weeks) were purchased from Charles River Technology and maintained and treated under specific pathogen-free conditions. To establish CRC xenografts, mice were subcutaneously injected with tumor cells (HCT-116, 1× 10^7^) in the back of the hind limbs of the nude mice. The mice were randomized into vehicle (*n* = 6) and treated groups (5 mg/kg and 10 mg/kg, *n* = 8 per group) after implantation for 1 week. CTD (dissolved in 5% DMSO and 10% Tween-20 in PBS) treatment was performed by intraperitoneal administration of CTD once every 2 days for 4 consecutive weeks. The control group received vehicle only. Tumor volume was measured twice a week and calculated using the following formula: (length × width × depth × 0.52). The body weights were recorded every 3 days. Finally, after the mice were sacrificed, the xenograft tumors were removed and frozen in liquid nitrogen or fixed in 4% formalin and embedded in paraffin for further studies.

### Immunohistochemical analysis

Paraffin-embedded sections (7 μm) were prepared for immunohistochemistry (IHC) analysis. The tissue samples were deparaffinized, hydrated, and permeabilized with 0.5% Triton X-100 in 1× PBS for 10 mins, and the sections were then blocked with 1% BSA and incubated with primary antibodies (Ki-67, p-AKT (Ser473), and p53) at 4 °C overnight. Then, cells were washed thrice with PBS and incubated with appropriate secondary antibody. 3, 3′-Diaminobenzidine (DAB) staining was used to visualize the protein targets as per the manufacturer’s instructions. Finally, cells were counterstained with hematoxylin for 2 min and photographed using a microscope, followed by analysis and quantification using the Image-Pro Plus software (v.6.1) (Media Cybernetics).

### Statistical analysis

All quantitative results were expressed as mean values ± SD. Significant differences were compared using the two-tailed independent sample *t*-test between the different groups, and *p*-values < 0.05 were considered to be statistically significant.

## Results

### CTD effectively inhibits the proliferation of CRC cells

The traits of cancer are inappropriate cell proliferation. To determine the effects of CTD on the proliferation of CRC cells, the impact of CTD on the toxicity of healthy human colon cells (CCD-18Co) was first estimated (Fig. S1a), which was used to determine the proper dosages for subsequent experiments. Meanwhile, CTD significantly inhibits the cell viability of CRC cells (Fig. 1b) based on toxicity dosages of healthy cell compared with control. Through detailed statistical analysis (Fig. 1c), it is more likely that CRC cells are adversely influenced in response to CTD at a dose of 10 μM. Therefore, 10 μM was selected as the maximal concentration for the following experiments. Moreover, the anchorage-independent growth assay revealed that the number and size of colonies were significantly reduced significantly in the CTD-treated group than in the DMSO control group (Fig. 1d), which was consistent with its suppressive effects on cell proliferation. The representative image illustrates the number of colonies as shown in Fig. S1b.

### CTD triggers cell cycle arrest in the G2/M phase and apoptosis in CRC cells

To verify that the antiproliferative activity of CTD in CRC corresponds to cell cycle regulation, flow cytometry analysis was performed to check cell cycle and apoptosis. Consequently, treatment with CTD for 24 h, the cell cycle arrested at the G2/M phase in CRC cells and the expression of cyclin B was decreased as detected by western blotting (Fig. 1f). Moreover, the apoptosis rates were gradually increased in CRC cells with an increase in CTD dosage (Fig. 1g and Fig. S1d). Meanwhile, western blot showed that the expressions of p53, cleaved-caspase-3, and Bax were considerably upregulated in response to CTD while the expressions of Bcl-2 and MDM2 were downregulated (Fig. 1h), indicating that CTD could trigger apoptosis in CRC cells. All the above evidence illustrates that CTD could affect the proliferation of CRC cells.

### CTD efficiently inhibits the migration and invasion of CRC cells

**Fig. 2:**
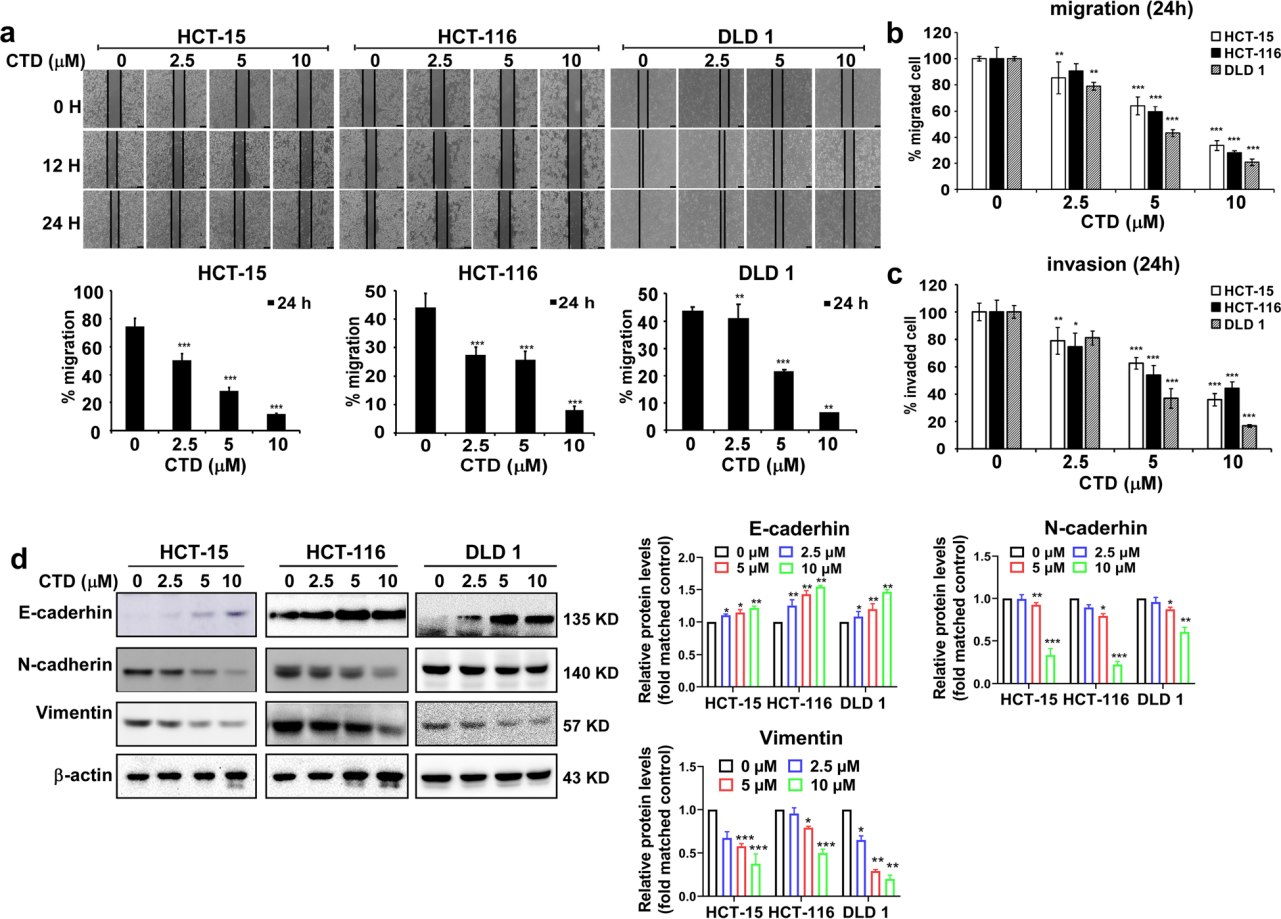
CTD inhibits the migration and invasion of colon cancer cells. A wound-healing assay was performed to measure the effect of CTD on the migration of the CRC cell lines at 24 h. **a** Treatment with CTD remarkably inhibited the migratory ability of the CRC cells in a wound-healing assay. Scale bars, 100 μm. The representative images (up) and the summary bar chart (down) of the cells that migrated by ImageJ to calculate the area. **b** and **c** Transmembrane migration assay was performed to measure the effect of CTD at various concentrations on the migration and invasion of the CRC cells at 24 h. This result indicated that CTD suppresses migration and invasion. The data are shown as means ± SD of values from triplicate samples. (*p < 0.05, **p < 0.01, ***p < 0.001) indicate a significant difference compared to the control. **d** Protein expression of epithelial-mesenchymal transition (EMT) markers E-cadherin, N-cadherin, and vimentin levels were checked after CTD treatment and compared with control

The migration and invasion are crucial in the initial progression of cancer that facilitates metastasis. Thus, we performed a wound-healing and transwell assays to investigate the function of CTD on migration and invasion. The wound-healing assay showed that CRC cells treated with CTD for 24 h migrated significantly slower than DMSO-treated control cells (Fig. 2a), suggesting that CTD could significantly dramatically decrease the migratory ability of cells. Furthermore, the effect of CTD on the migration and invasion ability of CRC cells was further investigated using the transwell assay. Compared with the control, the number of migrated and invaded CRC cells incubated with CTD significantly decreased (Fig. 2b-c and Fig. S2), indicating that CTD could dramatically reduce the migration and invasion abilities. Western blot analysis showed that E-cadherin was remarkably promoted following CTD treatment, and N-cadherin and vimentin were decreased (Fig. 2d), which are all markers of the EMT process in cancers. Therefore, these data imply that CTD inhibits colon cancer metastasis by suppressing migration and invasion.

### CTD inhibits the AKT-MDM2-p53 signaling pathway by binding and inactivating AKT in CRC cells

**Fig. 3:**
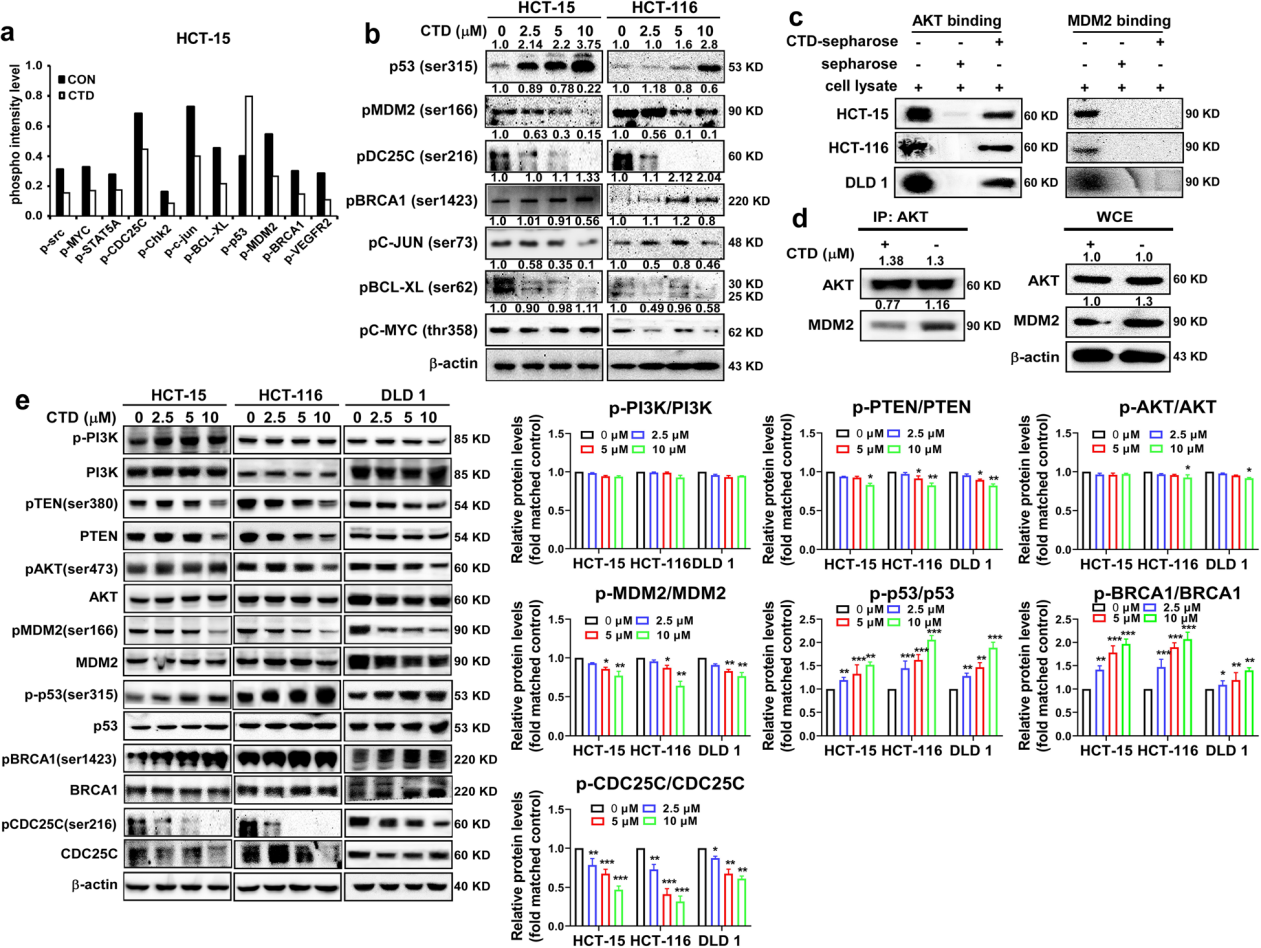
CTD regulates the AKT-MDM2-p53 signaling pathway. **a** Cancer signaling Phosphor Antibody Microarray was performed in HCT-15 cells and followed the CTD (10 μM) treated and untreated cells. **b** The microarray results were validated by western blotting. **c** CTD binds with AKT in the CRC cells. **d** HCT-116 cells were treated with or without 10 μM of CTD for 24 h. Cell lysates were immunoprecipitated with 1 μg of AKT and/or MDM2 antibodies, and binding activities were analyzed by western blot analysis. **e** The signaling pathway of AKT–MDM2–p53 was examined by the western blot analysis after CTD treatment for 24 h at different concentrations (0, 2.5, 5, or 10 μM) in the CRC cells

To gain mechanistic insights into the role of CTD in regulating colon cancer cell proliferation and apoptosis. Phospho-antibody microarray (PSAA) was conducted to identify the altered phosphorylated proteins following CTD treatment of HCT-15 cells. Consequently, altered in a series of phosphorylated proteins were associated with cell proliferation, cell cycle, and apoptosis, including MDM2, p53, CDC25C, BRCA1, and c-Myc. Western blot assay further confirmed these protein changes (Fig. 3a-b). Since MDM2 played a crucial role in regulating the stability of p53 and served as an excellent substrate for AKT, we speculate that CTD is likely to inhibit cell proliferation via the AKT-MDM2-p53 pathway in CRC.

Furthermore, to verify this purpose, the pull-down assay in vitro was performed using CTD-conjugated Sepharose 4B beads (or Sepharose 4B as a negative control) with cancer cell lysates. The results revealed that CTD was directly bound with AKT but not with MDM2 (Fig. 3c). Moreover, AKT interaction with MDM2 was examined by immunoprecipitation, and MDM2 expression was found to be suppressed after CTD treatment (Fig. 3d). Therefore, the findings of the PSAA and the binding ability of CTD with AKT showed the effects of CTD treatment on the AKT-MDM2-p53 signaling pathway in the CRC cells. The signaling pathway indicated that the activation of p53 was promoted by CTD treatment, while the expression levels of phosphorylated MDM2, a direct downstream of AKT, appeared to decrease. Furthermore, phosphorylation of BRCA1 and CDC25C were also altered, which were related observably to the event of DNA damage (Fig. 3e). Thus, we speculate that CTD induces apoptosis by activating p53 via the AKT/MDM2/p53 pathway or partially via DNA damage. Furthermore, our results indicate that p-AKT and AKT are considerably upregulated in the CRC tissues compared with others nearby (Fig. S3a-b) through the CRC tumor tissue microarray that contains 79 pairs of cancer and paracarcinoma tissues. The result was consistent with published microarray data (NCBI/GEO/GSE21815), the mRNA expression of AKT was much higher in the CRC tissues than the adjacent tissues (Fig. S3c). Although there was no significant difference in the overall survival between the high and low levels of AKT (*p* = 0.46) (Fig. S3d) (http://gepia.cancer-pku.cn/), AKT was highly expressed in cancer cells compared with normal cells (Fig. S3e) as well as in tumor tissue. These results indicated that AKT is a potential target in CRC.

### CTD promotes p53 expression by inhibiting MDM2 ubiquitination

**Fig. 4:**
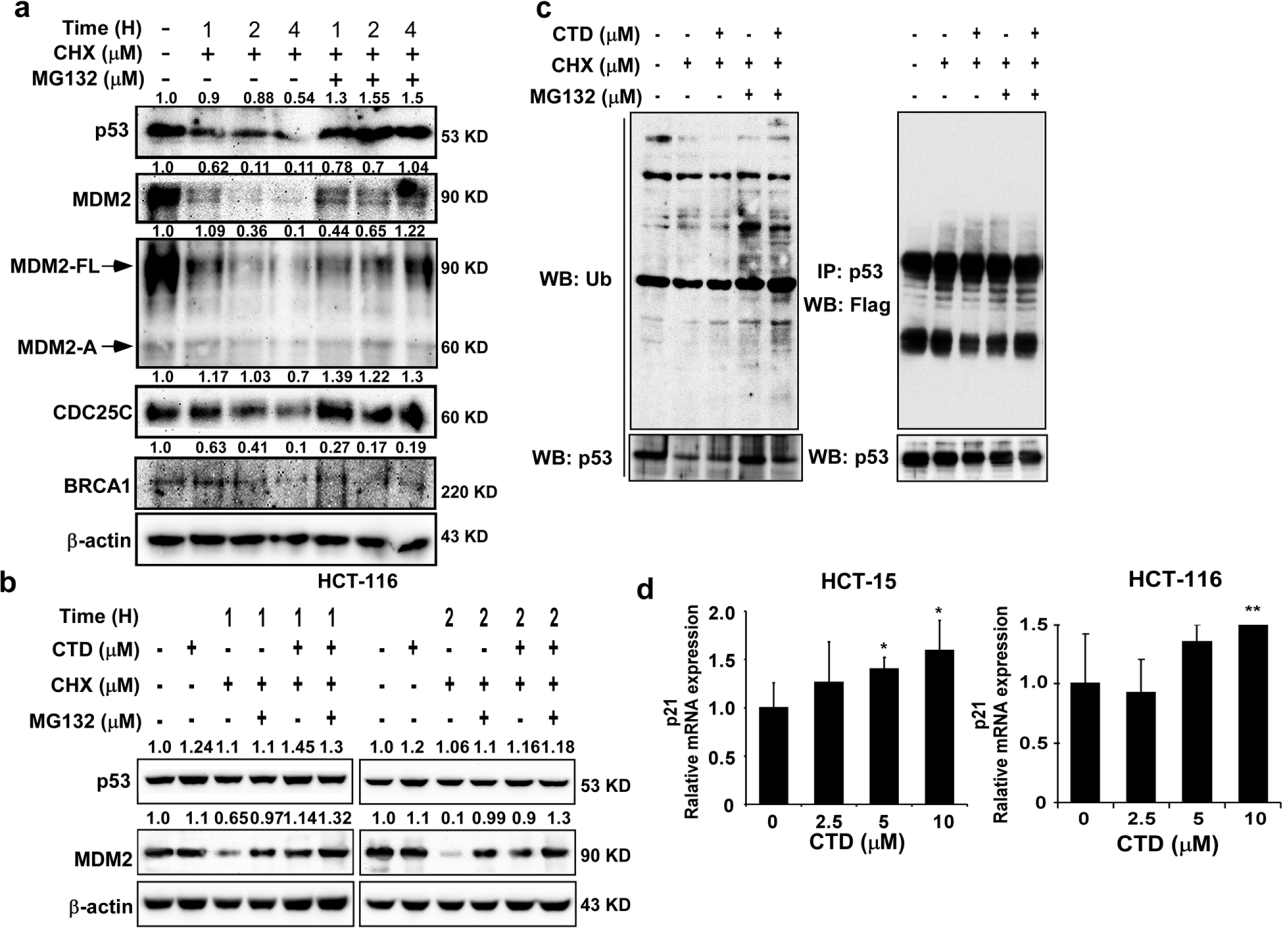
CTD promotes p53 expression by inhibiting MDM2 ubiquitination and upregulates its target. **a** Effect of the proteasome degradation pathway in the regulation of p53 stability. The HCT-116 cells were treated with cycloheximide (CHX) with or without MG132. p53 and the related protein were visualized by western blotting. **b** CTD induced the expression of p53 in the presence of CHX and MG132, respectively. This result indicated that p53 and MDM2 show a negative correlation. **c** CTD inhibited endogenous p53 ubiquitination. The HCT-116 cells were transfected with a 5 μg Flag-Ub, and the HCT-116 cells were treated with or without CTD, MG132, and CHX before harvesting as indicated. 24 h after transfection, the cell lysates were immunoblotted (left) and p53 was immunoprecipitated from 500 μg of cell lysate and immunoblotted with the antiubiquitin antibody to detect the ubiquitinated p53. **d** The CTD upregulated the transcriptional activity of p53. The mRNA levels of p21 are ascended, which is downstream of p53, and the indicated genes were analyzed by real-time quantitative PCR in the CRC cells by treated CTD. The data are shown as means ± SD of values from triplicate samples. (*p < 0.05, **p < 0.01, *** *p* < 0.001) indicate a significant difference between the untreated control and CTD-treated cells

A previous study has reported that the PI3K-AKT pathway inhibits p53-mediated transcription and apoptosis through enhanced MDM2-mediated ubiquitination [10]. Moreover, Sun et al. found that activation of the PI3/AKT/MDM2 pathway can promote the p53 degradation in the human hepatocellular cancer cells [15]. Therefore, to explore the potential mechanism underlying of MDM2-mediated regulation expression of p53 in the presence of CTD, we conducted protein stability assays. We co-treated HCT-116 cells with cycloheximide (CHX) and/or MG132 and found that the level of p53 protein gradual declined by CHX treatment, which was restored by co-treatment with MG132 and CHX (Fig. 4a). This result indicated that the protein stability of p53 might be mediated via the ubiquitin–proteasome protein degradation pathway. Moreover, similar phenomenon also appeared in MDM2. The p53 is a particular substrate of MDM2, which inhibits p53 by promoting its ubiquitination and degradation. Also, our results showed that MDM2 and p53 could regulate each other and showed a negative correlation in the presence of CHX and MG132 (Fig. 4b), and to prevent protein degradation, the ubiquitination assay of ex vitro by transfection of cancer cells with FLAG-tagged ubiquitin showed that p53 ubiquitination was decreased by co-treatment with CTD and MG132 (Fig. 4c). The results suggested that CTD could prevent MDM2-mediated ubiquitination of P53 and its degradation. Since p53 will be activated by the inhibition of MDM2 after CTD treatment, RT-PCR was performed to confirm the downstream effects of p53, which showed that p21 was increased. Thus, CTD induced apoptosis through the activation of p53 transcription (Fig. 4d).

### CTD affects the intracellular localization of MDM2 and p53

**Fig. 5:**
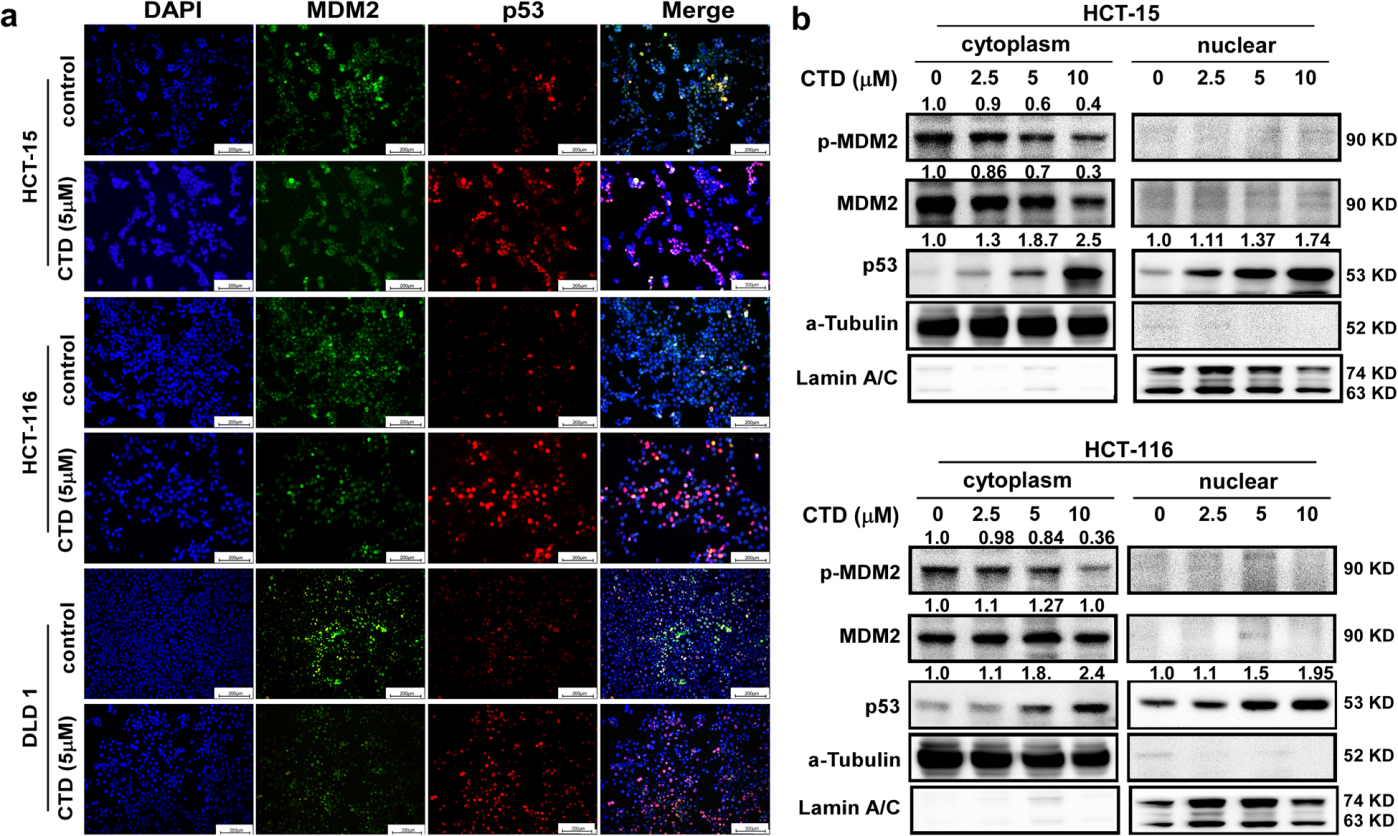
CTD affects the intracellular localization of MDM2 and p53 and p53 activity. **a** Effects of CTD on the intranuclear content of p53 and MDM2. The cells were stained for p53 (red) and MDM2 (Green), and the nuclei were counterstained with DAPI (blue). The localization of p53 and MDM2 expressions in the cells is indicated in the merged image. Scale bar = 200 μm. **b** p53 was upregulated, and MDM2 was suppressed and translocated to the nucleus following the CTD treatment. The endogenous p53 and MDM2 were detected in the cytoplasm and nucleus of CRC cells treated with CTD for 24 h using an anti-p53, MDM2, tubulin, and lamin A/C antibodies for western blotting

The stabilization of p53 by preventing the nuclear-to-cytoplasmic movement of MDM2 or by inhibiting the intrinsic E3 ubiquitin ligase activity of MDM2 is the primary mechanism by which MDM2 affects p53. Furthermore, to examine the subcellular expression and localization of the p53 and MDM2 axis following CTD treatment. The immunofluorescence staining assay was performed, and the p53 was demonstrated to be upregulated and accumulated in the nucleus by the treatment with CTD at a dose of 5 μM (Fig. 5a), which is consistent with the results of western blot analysis with separated nuclear and cytoplasmic proteins (Fig. 5b). Meanwhile, the expression levels of MDM2 in the cytoplasmic and nuclear fractions appeared to decrease after CTD treatment (Fig. 5a-b). Thus, p53 accumulation was mediated by CTD via inhibition of MDM2. These findings suggest that CTD treatment promotes p53 transcriptional activity.

### Anticancer activity of CTD is dependent on the expression of AKT

**Fig. 6:**
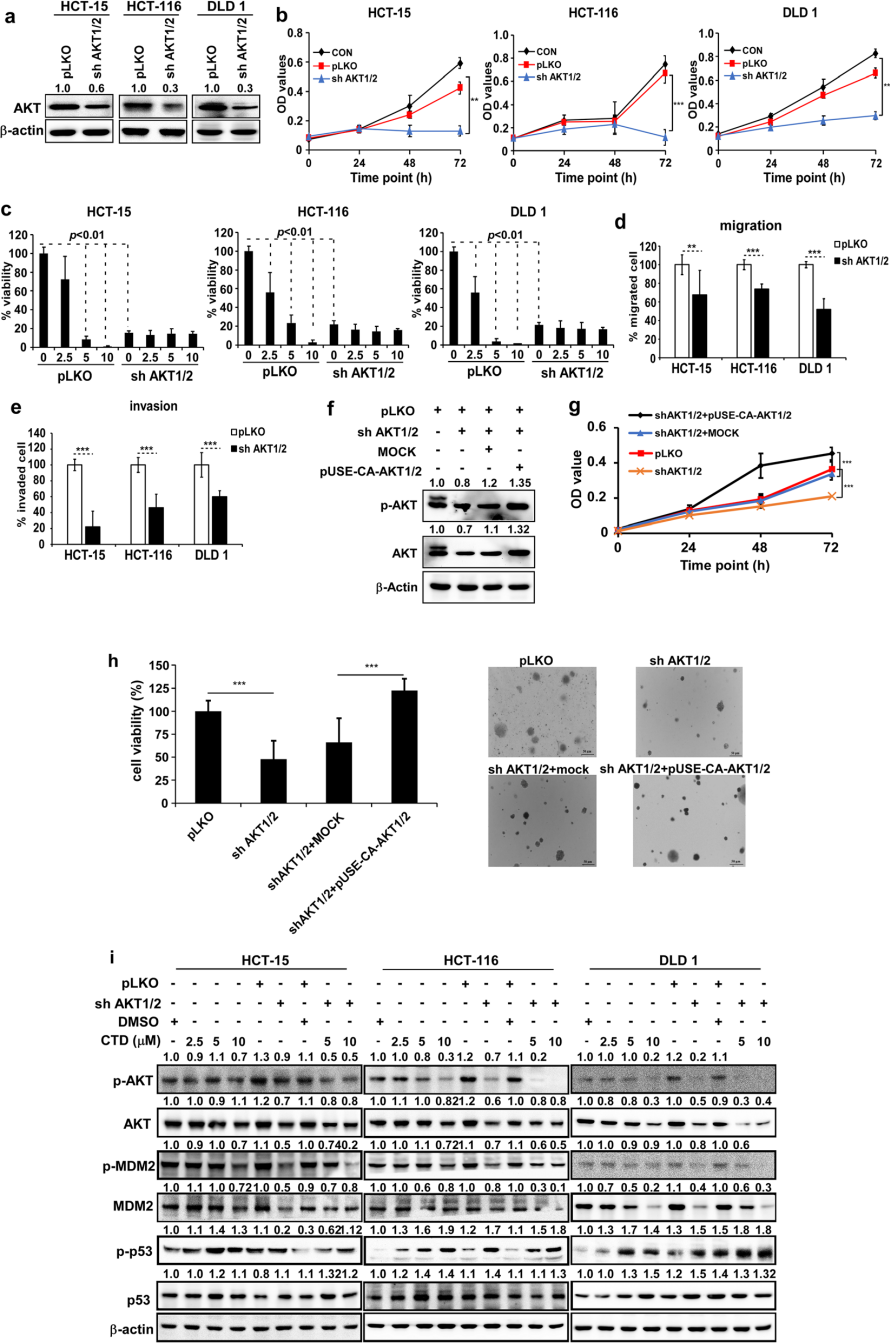
AKT is a therapeutic target in the CRC cells. **a** Effect of the knockdown of AKT1/2 on total AKT protein. The CRC cells stably expressing AKT1/2 knockdown or control were established. The expression was determined by western blotting. **b** The cell growth in the CRC cells after AKT1/2 knockdown by the CCK-8 assay at different time points. **c** The effect of CTD treatment on the anchorage-independent growth was assessed in the CRC cells expressing pLKO or shRNA-AKT1/2. The anchorage-independent cell growth is decreased with or without CTD treatment after AKT1/2 expression was decreased. **d** The ability of migration was detected by the transwell assay after the knockdown of the AKT1/2. **e** The ability of invasiveness was detected by the transwell assay after the knockdown of the AKT1/2. **f** The AKT expression level of pLKO, shRNA-AKT1/2, overexpressed pUSE-CA-Akt1/2 and mock by western blot analyzed. **g** and **h** The proliferation of cells expressing pLKO or shRNA-AKT1/2 or overexpressing pUSE-CA-Akt1/2 and corresponding mock control were examined by the MTT and anchorage-independent cell growth assays. All the data are shown as means ± SD of values from triplicate samples. (*p < 0.05, **p < 0.01, *** *p* < 0.001) indicate a significant difference compared to the control. **i** The additive effects were observed via the coordinated inhibition of MDM2-p53 interaction and AKT, as shown by immunoblotting. MDM2, p53, and AKT were evaluated by immunoblotting after treatment with CTD for 24 h and infection with lentiviral AKT shRNA for 24 h. Actin is used as a loading control

Based on the results of the binding assay and the AKT expression being high in CRC. To investigate whether the inhibition of CTD on cell proliferation is dependent on the AKT protein expression, knockdown of AKT was performed by infecting the cells with virus particles containing pLKO or shRNA-AKT1/2 (Fig. 6a), which we used for the CCK-8 and anchorage-independent cell growth assays. The knockdown of AKT1/2 in the CRC cells resulted in decreased cell proliferation and colony formation compared to the pLKO-infected cells (Fig. 6b-c). Furthermore, we confirmed the effects of CTD on cell proliferation by targeting AKT1/2. The results demonstrated that treatment with CTD failed to further suppress the colony formation in the shRNA-AKT1/2 cells (Fig. 6c and Fig. S4a). Also, we found that migration and invasion were suppressed after AKT1/2 knockdown (Fig. 6d-e and Fig. S4b). Immunofluorescence staining showed that p53 was activated after AKT1/2 knockdown in the CRC cell lines compared with the pLKO group (Fig. S5). Besides, rescuing AKT1 or AKT2 expression in AKT1/2 knockdown cells restored cell growth, as indicated by the MTT and anchorage-independent cell growth assays (Fig. 6f-h). Moreover, AKT1/2 overexpressing cells showed higher sensitivity to CTD than the mock control (Fig. S6). Finally, the AKT-MDM2-p53 pathway was analyzed after the AKT knockdown by western blotting. Consequently, p53 was increased after the AKT was silenced (Fig. 6i). The results indicated that AKT is a target of CTD, which can suppress cell proliferation, migration, and invasion via the activation of p53.

### CTD regulates the antiproliferative activity and apoptotic levels in a p53-independent manner

**Fig. 7:**
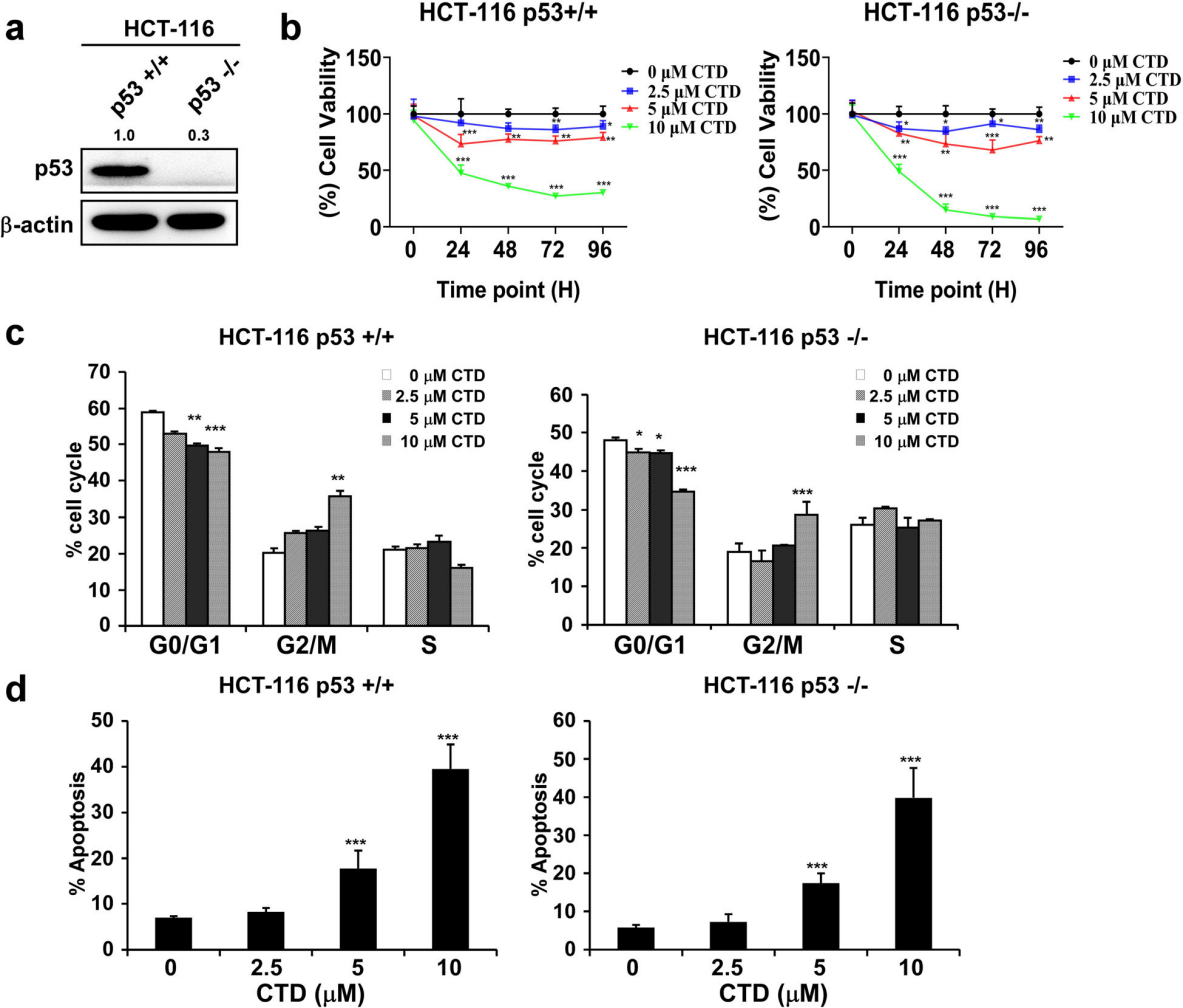
CTD inhibits colon cancer cell growth irrespective of cellular p53 status. **a** The expression of p53 in these two cell lines by western blot. **b** The growth of HCT-116 p53+/+ and p53−/− cells were determined by the MTT assay after treatment with various CTD concentrations (0, 2.5, 5, or 10 μM) for 24, 48, 72, or 96 h, and similar results were obtained. **c** The effect of treatment with CTD on HCT-116 p53+/+ and p53−/− cells was evaluated. **d** The effect of CTD on apoptosis was assessed in HCT-116 p53+/+ and p53−/− cells. All the data are shown as means ± SD of values from triplicate samples. (*p < 0.05, **p < 0.01, *** *p* < 0.001) indicate a significant difference compared to the control

To address whether the antiproliferative and apoptotic effects of CTD on CRC cells are dependent on intracellular p53, we first confirmed the expression of p53 in HCT-116 53+/+ and HCT-116 p53−/− cells (Fig. 7a). Then, HCT-116 p53+/+ (wild type) and HCT-116 p53−/− (deficient) cells were treated with different concentrations of CTD for 24, 48, 72, and 96 h. The results revealed that the proliferation of the two cell lines was significantly inhibited after CTD treatment (Fig. 7b). Cell cycle was arrested at the G2/M phase in both cell types irrespective of p53 status (Fig. 7c).

Treatment with CTD induced apoptosis in the two cell lines (Fig. 7d). Summarily, CTD inhibits colon cancer cell growth independent on p53 statues.

### CTD inhibits the growth of the xenografts model

**Fig. 8:**
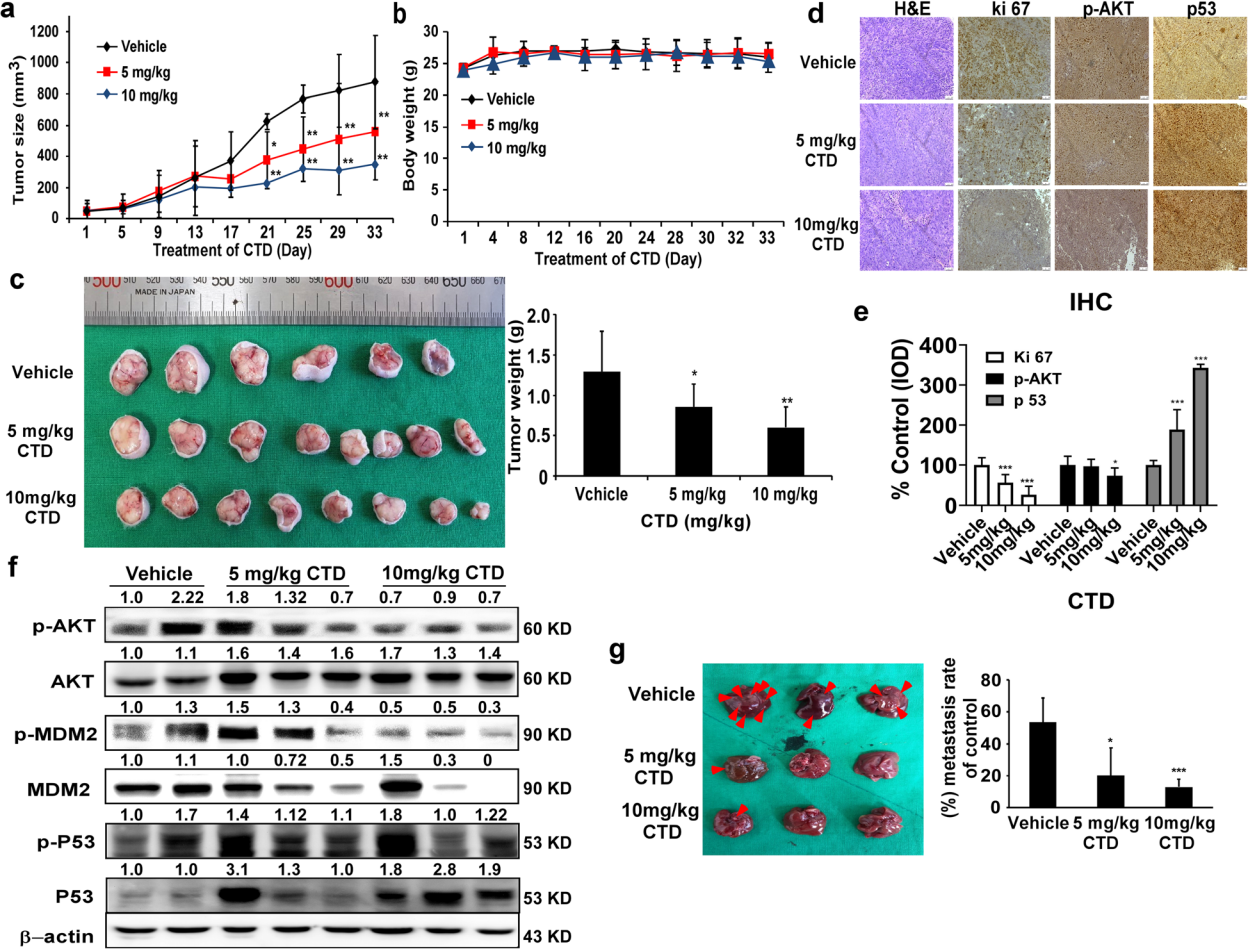
CTD inhibits tumor growth in vivo. **a** The effect of CTD on the volume of CDX tumors was plotted over 30 days (HCT-116). Vehicle and CTD (5 or 10 mg/kg) were administered intraperitoneally. The tumor volume was measured twice a week. **b** The body weight was stable after CTD treatment indicating a lack of toxicity at these doses. **c** The photographs (left) show tumors from CDX mice treated with vehicle or CTD (5 or 10 mg/kg), and the weights of the tumors were quantified and expressed as the treatment groups compared with the vehicle-treated group (right). **d** The expressions of Ki-67, p-AKT, and p53 were examined by IHC analysis (100× magnification). **e** The quantitation of the protein expression from IHC staining. The values are quantified from IHC staining and expressed as the treatment groups compared with the vehicle-treated control group. The data are expressed as IOD. **f** CTD affects the expression of AKT-MDM2-p53 signaling proteins in the CDX tumor tissues. The tumor tissues were analyzed by western blotting. **g** Left, the representative images of the excised livers after the mice were sacrificed (arrows indicate the metastatic nodules). Right, the graph showing the number of surface metastatic foci in the livers. All data are shown as the mean value ± SD. (*p < 0.05, **p < 0.01, *** *p* < 0.001) indicate a significant decrease compared to the control

Finally, our objective was to confirm the anticancer activity in vivo. We implanted the HCT-116 cells in athymic nu/nu mice for 1 week and then treated the mice with 5 mg/kg or 10 mg/kg of CTD once a day, twice a week for four consecutive weeks. Consequently, both of these tested doses decreased the tumor volume and weight compared to the vehicle group (Fig. 8a-c), with no effect on the body weight (Fig. 8b). The tumor tissues were prepared for immunohistochemical analysis to detect the expression of ki-67, p-AKT, and p53 and showed that all of these protein markers were remarkably ameliorated by the CTD treatment compared with the vehicle (Fig. 8d-e). Compared with the immunoblotting analysis of the cell lysates from the xenograft tumors showed that CTD significantly changed the state of p-AKT, p-MDM2, and p53 protein expressions compared to the vehicle (Fig. 8f). In addition, to determine the potential toxic effects of CTD on tissue morphology, the liver, spleen, and lung tissues from the CDX mice were analyzed by hematoxylin and eosin staining, which showed no morphological changes between the CTD-treated and vehicle-treated groups. Our results also showed that CTD remarkably reduced the ability of liver metastasis in nude mice compared with the vehicle treatment, evidenced by the smaller number of metastatic tumor nodules in the livers (Fig. 8g).

## Discussion

There are multiple effects for the anticancer activity of CTD. However, the precise and primary molecular targets of CTD remain undetermined. Although prior studies showed the CTD play a crucial role in colon cancer, breast cancer, and antineuroinflammation through target thioredoxin reductase, detyrosinated tubulin, and CDK2, respectively [26, 32, 33], we found that CTD could inhibit the proliferation of cancer cells by directly targeting AKT expression. Studies also show that CTD inhibits the differentiation and represses hepatic fibrosis by regulating mitogen-activated protein kinases and Notch3 degradation, respectively. Or it suppresses the colon cancer growth by the Wnt/β-Catenin pathway [34–36]. However, our findings suggest that CTD regulates MDM2/p53 interaction by inhibiting AKT activation, which thereby increase apoptosis in cancer cells. Obviously, CTD is a nonselective, polytargeted drug.

The AKT was considered to be an attractive target for cancer therapy and prevention. It was reported that AKT acts as a target in many cancers [37]. However, AKT as a target of colorectal cancer has been rarely reported, even though some inhibitors have been used for CRCs. In this study, we found that AKT was highly expressed in the CRC tissues and CRC cell lines compared with normal control (Fig. S3). Besides, knockdown of AKT1/2 remarkably suppressed the growth of the CRC cells (Fig. 6), which are consistent with the previous reports [38]. Notably, cell growth failed to increase after CTD treatment in the knockdown cell of AKT1/2, and AKT1/2 restored the cell growth in the rescue process (Fig. 6c, f-h). Our results demonstrate that CTD suppresses AKT-induced growth in the in vivo models of CRC. Thus, we considered that CTD acted as an AKT inhibitor to suppress the growth of CRC.

The CTD was reported to inhibit cell proliferation in cancer cell lines at different doses [30], including colon cancer cells. Zhuge et al. [26] reported that CTD induced apoptosis at 30 μM in the colon cancer cells. Contrastively, we found that CTD induced G2/M phase arrest and apoptosis at 10 μM, whereas have no effects on the CCD-18Co growth of the healthy colon cells within the dose range (Fig. 1 and Fig. S1a). Moreover, a previous study reported that CTD suppresses metastasis at a lower concentration in osteosarcoma cells [39], which matches our migration and invasion assay findings (Fig. 2). The above results indicate that CTD has intense antiproliferative activity on colon cancer cells. Mechanistically, CTD was reported to induce apoptosis via activate ROS and mitochondrial pathway. Recent studies have revealed that CTD treatment activates p53, leading to an inhibition of proliferation and cell cycle arrest [40, 41], indicating that p53 plays an essential role in anticancer progress. Our PSAA results showed that p53 expression was induced after CTD treatment, and related proteins, such as MDM2, were also changed (Fig. 3b). Furthermore, some studies have suggested that the changes in MDM2 expression affect the balance of p53 signaling [42–44], which ultimately influences tumorigenesis. However, the MDM2-mediated p53 degradation was not explicitly established in vivo, whose interaction seems “druggable” using a variety of strategies [45]. In this study, we found that targeting AKT with CTD inhibition of cell growth via the AKT-MDM2-p53 pathway is an effective way to treat cancer.

Moreover, we found that CTD could inhibit the interaction of AKT and MDM2 compared with the absence of CTD (Fig. 3c). Therefore, we considered that CTD could play an anticancer role via p53 activation by inhibiting AKT-mediated MDM2 phosphorylation. Besides, Yoko et al. reported that AKT enhanced MDM2-mediated ubiquitination and degradation of p53 [11]. However, from our study, CTD could prevent the MDM2-mediated ubiquitination of p53 (Fig. 4c). Observably, the underlying mechanisms of CTD on cell proliferation may be associated with regulation of p53 ubiquitination and decrease degradation of p53 by targeting AKT, which thereby promoting the expression of p53. In addition, p21 expression induced indicated activation of p53 under the treatment of CTD (Fig. 4d). Furthermore, via immunofluorescence staining and extraction of cytosolic and nuclear fractions, our results clearly demonstrate that CTD potently induces nuclear accumulation of p53 (Fig. 5a). Thus, our data confirm the hypothesis that CTD -induced stabilization and nuclear accumulation of p53 induces an increase in its transcriptional activity and results in an upregulation of its target gene p21.

The results of PSAA also demonstrated that the downstream of ATM is BRCA1 and CDC25C were also altered, which is associated with DNA damage and cell cycle. Earlier studies demonstrated showed that the transcriptional activity of p53 was enhanced by phosphorylation of p53 by ATM [46, 47]; while phosphorylation of MDM2 by ATM impaired the ability of MDM2 to promote p53 degradation, which means that BRCA1 and p53 may simultaneously contribute to induce apoptosis. More so, the expression of phosphorylation CDC25C was modulated, which may lead to G2/M arrest of CTD-treated cells, which is in line with previous breast cancer reports [48]. Besides, LE and co-workers have reported that MDM2 interacts with CDC25C in a p53-independent manner promotes its degradation in an ubiquitin-independent way in lung cancer [49]. Therefore, CTD probably activates the p53 pathway and sensitizes colon carcinoma cells to the cytotoxic effects of CTD at a lower concentration. Moreover, c-MYC, as another apoptosis gene driver, regulates the level and activity of p53 in a dependent [50] (response to DNA damage) or nondependent manner [51] (downregulation of AKT1). Therefore, the c-MYC function is a requirement during the apoptosis induction following DNA damage. Thus, targeting AKT with CTD may partially induce apoptosis via DNA damage, in contrast to previous reports stating that AKT fails to cause DNA damage [17]. Together, these data demonstrate that in addition to the known p53-dependent mechanism, CTD can activate p53-independent pathways, such as AKT-p21 signaling [52], leading to apoptosis (Fig. 7).

Finally, the xenograft model was conducted to ascertain the anticancer effects of the CTD against colon cancer in vivo, which is valid for the preclinical findings that provide valuable directives into the mechanism [53, 54]. Compared with the vehicle-treated mice, intraperitoneal CTD injection significantly decreased the tumor growth (Fig. 8a-c) without toxicity based on no bodyweight loss, even in the liver, spleen, and H&E staining analysis compared with the vehicle-treated mice. The xenograft tumors we analyzed via IHC indicated that CTD mitigated Ki-67, p-AKT and promoted p53 expression in CDX models (Fig. 8d-e), suggesting that CTD activates p53 expression by targeting AKT. Additionally, our results also showed that CTD could suppress tumor metastasis (Fig. 8g). All of which indicating the potential of developing CTD as a novel inhibitor of AKT for CRC.

## Conclusions

**Fig. 9:**
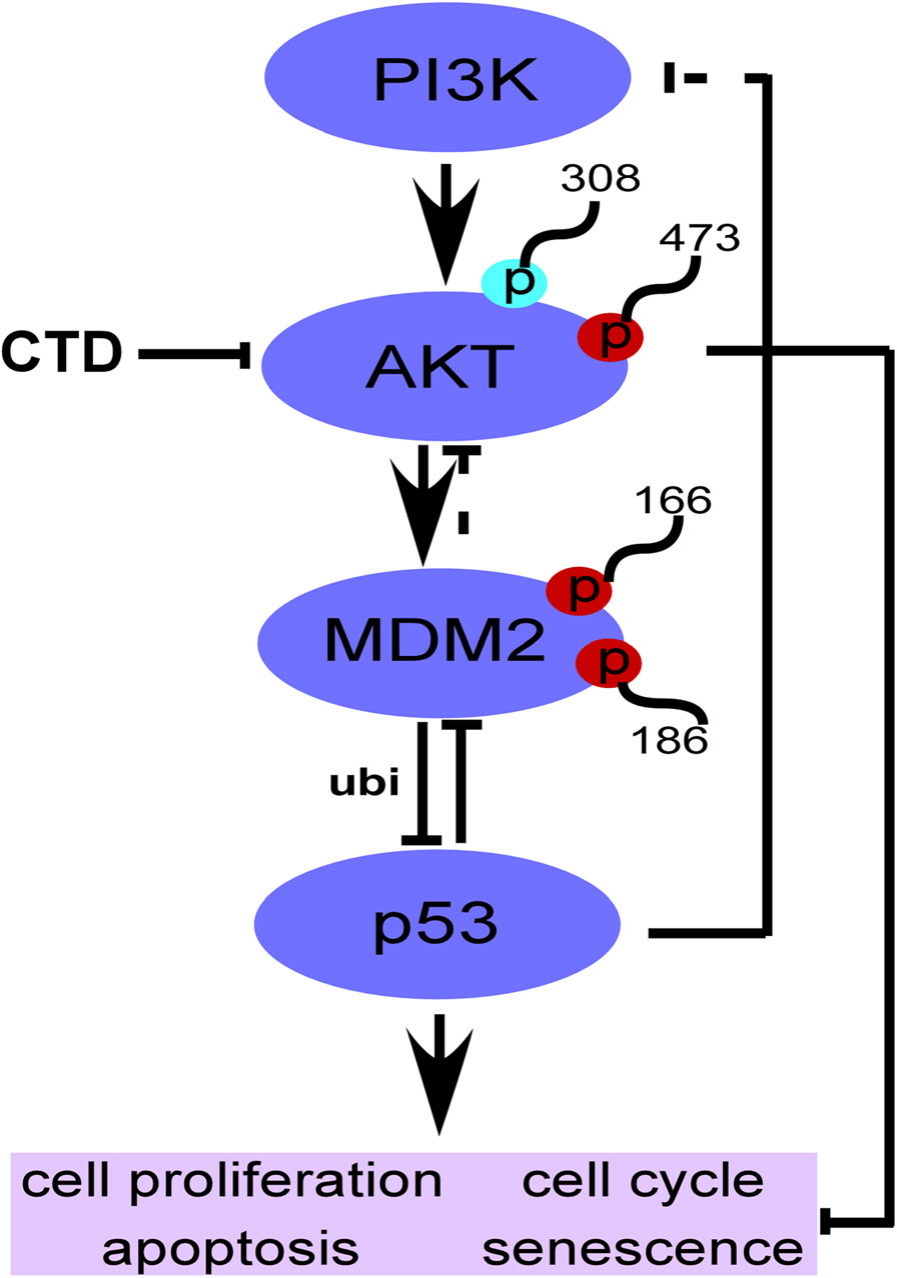
A proposed scheme illustrating the distinct roles of AKT1/2 in CRC and their modulation by CTD. AKT is activated by upstream kinases. Receptor tyrosine kinase (RTK) or PI3K stimulates AKT, thereby promoting cell proliferation and the cell cycle, preventing apoptosis. Targeting AKT with CTD exerts its potential anticancer effect to induce cell cycle arrest and apoptosis and inhibit cell proliferation through the dependent or independent AKT pathway

Our study identified that p53 was activated upon CTD treatment to execute its anticancer activity via the AKT/MDM2/p53 pathway and that CTD suppresses the proliferation of human CRC cells by inhibiting MDM2-mediated p53 ubiquitination and degradation by targeting AKT to some extent. Therefore, the activation of the formidable p53 tumor suppressor function by suppressing the interaction of MDM2–p53 has being a new cancer therapeutic strategy [55–57]. Meanwhile, we cannot ignore the natural compounds contribution to these inhibitors derived from edible and medicinal plants, which have shown potential chemopreventive and chemotherapeutic activities in preclinical and clinical studies [58]. Finally, the spotlight falls back on our compound that suppresses colon cancer cells in vitro and in vivo by targeting AKT (Fig. 9), suggesting that AKT represents an attractive target and CTD also can be developed as an AKT inhibitor for CRC chemotherapy.

## Supplementary Information

**Additional file 1: Figure S1.** Analysis of cell proliferation, cell cycle, and apoptosis after treatment with CTD. (a) MTT assay was performed to measure the effect on the cytotoxicity of healthy colon cells named CCD-18Co at 24, 48, 72, and 96 h, respectively. Data are shown as mean ± SD of values from triplicate samples. (*p < 0.05, **p < 0.01, ***p < 0.01) indicate a significant difference compared to control. (b) Corresponding images of anchorage-independent cell growth (magnification, 100×). (c) Representative photographs of the cell cycle after CTD treatment in CRC cells. Cell cycle was measured by flow cytometry in HCT-15, HCT-116, and DLD1 cells after treatment with various costunolide concentrations (0, 2.5, 5, or 10 μM) (d) Representative photographs of apoptosis after CTD treatment in CRC cells. Apoptosis was detected by flow cytometry in HCT-15, HCT-116, and DLD1 cells after treatment with different dose of Costunolide (0, 2.5, 5, or 10 μM).

**Additional file 2: Figure S2.** CTD suppressed the migration of colon cancer cells by a transmembrane assay. (a) Representative images of the in vitro migration assays in the transwell system. (b) Representative images of the in vitro invasion assay in the transwell system.

**Additional file 3: Figure S3.** AKT is frequently overexpressed in CRC. (a). The expression of phosphorylated AKT (Ser473) and total AKT (pan) was examined by IHC analysis using a CRC tumor microarray (100× magnification). The top panels show the quantitation of all samples. (b). Representative images of IHC staining on a CRC tumor microarray are shown. The AKT and p-AKT expression are stained in brown, and the nuclei are stained blue with hematoxylin. Two images are shown from each group (magnification, 100×). (c). The mRNA level of of AKT1, AKT2, and AKT3 expression was calculated from the data set GSE21815 (*N* = 123 pairs, which were examined using cDNA microarray from primary CRC and paired normal tissues). (*p < 0.05, **p < 0.01, ***p < 0.001) indicate a significant difference compared to control. (d). The overall survival time of patients with high or low AKT expression (http://gepia.cancer-pku.cn/). (e). Western blot analysis in one normal colon cell line (CCD-18 Co) and other normal skin (HaCaT) and six other types of cancer cell lines (colon cancer, lung cancer, breast cancer, liver cancer, prostate cancer, and melanoma).

**Additional file 4: Figure S4.** Confirm the AKT is the target of CTD in knockdown cells. (a). Representative colony pictures after AKT1/2 knockdown with or without CTD treatment. (b). Representative images of in vitro migration and invasion assays in the transwell system after the knockdown of AKT1/2.

**Additional file 5: Figure S5.** Effects of AKT1/2 knockdown on the intranuclear content of p53 and MDM2. The cells were stained for p53 (red) and MDM2 (Green), and the nuclei were counterstained with DAPI (blue). Localization of p53 and MDM2 expression in the cells are shown in the merged image. Scale bar = 100 μm.

**Additional file 6: Figure S6.** AKT is a therapeutic target of CRC cells. (a). Overexpression of AKT1/2 was detected by Western blot in CRC cell lines. (b). The effect of proliferation by MTT assay at different time points in CRC cell lines after overexpression of AKT1/2. (c). Anchorage-independent cell formation was assessed after treatment with different doses of CTD in cells expressing mock or AKT1/2. (d). Representative colony images after AKT1/2 over-expression with CTD treatment. (e). The quantification showing the effect of migration by transmembrane assay after overexpression of AKT1/2. (f). The quantification showing the effect of invasion by the transmembrane assay after overexpression of AKT1/2. (g). Representative images of in vitro migration and invasion assays in the transwell system. Data are shown as mean ± SD of values from triplicate samples. (*p < 0.05, **p < 0.01, ***p < 0.001) indicate significant differences compared to control.

## Data Availability

Supplemental Figures (1, 2, 3, 4, 5, and 6), and associated figure legends are provided as supplemental material and are available online with the paper. The RNA expression in colon cancer and the patients’ survival data generated in this study are available from Gene Expression Profiling Interactive Analysis (GEPIA http://gepia.cancer-pku.cn/).

## Abbreviations

CRC: Colorectal cancer
VEGF: Vascular endothelial growth factor
EGFR: Epidermal growth factor receptor
ROS: Reactive oxygen species
PI3K: Phosphoinositide-3 kinase
AKT: V-akt murine thymoma viral oncogene homolog
MDM2: Mouse double minute 2
CDX model: Cell-derived xenograft model

## References

[1] Siegel RL, Miller KD, Jemal A (2020). Cancer statistics, 2020. CA Cancer J Clin.

[2] Schmoll HJ, Tabernero J, Maroun J, de Braud F, Price T, Van Cutsem E, Hill M, Hoersch S, Rittweger K, Haller DG (2015). Capecitabine plus oxaliplatin compared with fluorouracil/folinic acid as adjuvant therapy for stage III colon cancer: final results of the NO16968 randomized controlled phase III trial. J Clin Oncol.

[3] Sasaki T, Kuniyasu H, Luo Y, Kitayoshi M, Tanabe E, Kato D, Shinya S, Fujii K, Ohmori H, Yamashita Y (2013). Increased phosphorylation of AKT in high-risk gastric mucosa. Anticancer Res.

[4] Liu X, Song M, Wang P, Zhao R, Chen H, Zhang M, Shi Y, Liu K, Liu F, Yang R (2019). Targeted therapy of the AKT kinase inhibits esophageal squamous cell carcinoma growth in vitro and in vivo. Int J Cancer.

[5] Zhu YP, Dai B, Zhang HL, Shi GH, Shen YJ, Ye DW (2016). Long non-coding RNA LOC572558 inhibits bladder cancer cell proliferation and tumor growth by regulating the AKT-MDM2-p53 signaling axis. Cancer Lett.

[6] Tu YY, Kim E, Gao Y, Rankin GO, Li B, Chen YC (2016). Theaflavin-3, 3′-digallate induces apoptosis and G2 cell cycle arrest through the Akt/MDM2/p53 pathway in cisplatin-resistant ovarian cancer A2780/CP70 cells. Int J Oncol.

[7] Choi HJ, Chung TW, Kang SK, Lee YC, Ko JH, Kim JG, Kim CH (2006). Ganglioside GM3 modulates tumor suppressor PTEN-mediated cell cycle progression--transcriptional induction of p21(WAF1) and p27(kip1) by inhibition of PI-3K/AKT pathway. Glycobiology.

[8] Jabbarzadeh Kaboli P, Salimian F, Aghapour S, Xiang S, Zhao Q, Li M, Wu X, Du F, Zhao Y, Shen J (2020). Akt-targeted therapy as a promising strategy to overcome drug resistance in breast cancer - a comprehensive review from chemotherapy to immunotherapy. Pharmacol Res.

[9] Ray RM, Bhattacharya S, Johnson LR (2011). Mdm2 inhibition induces apoptosis in p53 deficient human colon cancer cells by activating p73-and E2F1-mediated expression of PUMA and Siva-1. Apoptosis.

[10] Abraham AG, O'Neill E (2014). PI3K/Akt-mediated regulation of p53 in cancer. Biochem Soc Trans.

[11] Ogawara Y, Kishishita S, Obata T, Isazawa Y, Suzuki T, Tanaka K, Masuyama N, Gotoh Y (2002). Akt enhances Mdm2-mediated ubiquitination and degradation of p53. J Biol Chem.

[12] Matsuda S, Nakagawa Y, Kitagishi Y, Nakanishi A, Murai T (2018). Reactive oxygen species, superoxide dimutases, and PTEN-p53-AKT-MDM2 signaling loop network in mesenchymal stem/stromal cells regulation. Cells.

[13] Drakos E, Atsaves V, Li J, Leventaki V, Andreeff M, Medeiros LJ, Rassidakis GZ (2009). Stabilization and activation of p53 downregulates mTOR signaling through AMPK in mantle cell lymphoma. Leukemia.

[14] Mayo LD, Donner DB (2002). The PTEN, Mdm2, p53 tumor suppressor-oncoprotein network. Trends Biochem Sci.

[15] Sun WC, Tang LL (2016). MDM2 increases drug resistance in cancer cells by inducing EMT independent of p53. Curr Med Chem.

[16] Harris SL, Levine AJ (2005). The p53 pathway: positive and negative feedback loops. Oncogene.

[17] Astle MV, Hannan KM, Ng PY, Lee RS, George AJ, Hsu AK, Haupt Y, Hannan RD, Pearson RB (2012). AKT induces senescence in human cells via mTORC1 and p53 in the absence of DNA damage: implications for targeting mTOR during malignancy. Oncogene.

[18] Cao Z, Xue J, Cheng Y, Wang J, Liu Y, Li H, Jiang W, Li G, Gui Y, Zhang X (2019). MDM2 promotes genome instability by ubiquitinating the transcription factor HBP1. Oncogene.

[19] Wen W, Peng C, Kim MO, Jeong CH, Zhu F, Yao K, Zykova T, Ma W, Carper A, Langfald A (2014). Knockdown of RNF2 induces apoptosis by regulating MDM2 and p53 stability. Oncogene.

[20] Wang SM, Zhao YJ, Aguilar A, Bernard D, Yang CY (2017). Targeting the MDM2-p53 protein-protein interaction for new cancer therapy: progress and challenges. Cold Spring Harb Perspect Med.

[21] He SL, Wang WP, Yang YS, Li EM, Xu LY, Chen LQ (2019). FAM3B promotes progression of oesophageal carcinoma via regulating the AKT-MDM2-p53 signalling axis and the epithelial-mesenchymal transition. J Cell Mol Med.

[22] Fenouille N, Puissant A, Tichet M, Zimniak G, Abbe P, Mallavialle A, Rocchi S, Ortonne JP, Deckert M, Ballotti R (2011). SPARC functions as an anti-stress factor by inactivating p53 through Akt-mediated MDM2 phosphorylation to promote melanoma cell survival. Oncogene.

[23] Jin M, Park SJ, Kim SW, Kim HR, Hyun JW, Lee JH (2017). PIG3 regulates p53 stability by suppressing its MDM2-mediated ubiquitination. Biomol Ther.

[24] Zeng KX, Chen XX, Hu XX, Liu XX, Xu T, Sun HL, Pan YQ, He BS, Wang SK (2018). LACTB, a novel epigenetic silenced tumor suppressor, inhibits colorectal cancer progression by attenuating MDM2-mediated p53 ubiquitination and degradation. Oncogene.

[25] Holzer P (2017). Discovery of potent and selective p53-MDM2 protein-protein interaction inhibitors as anticancer drugs. Chimia.

[26] Zhuge W, Chen R, Vladimir K, Dong X, Zia K, Sun X, Dai X, Bao M, Shen X, Liang G (2018). Costunolide specifically binds and inhibits thioredoxin reductase 1 to induce apoptosis in colon cancer. Cancer Lett.

[27] Peng ZX, Wang Y, Fan JH, Lin XJ, Liu CY, Xu Y, Ji WD, Yan C, Su CQ (2017). Costunolide and dehydrocostuslactone combination treatment inhibit breast cancer by inducing cell cycle arrest and apoptosis through c-Myc/p53 and AKT/14-3-3 pathway. Sci Rep.

[28] Hua PY, Sun M, Zhang GX, Zhang YF, Song G, Liu ZY, Li X, Zhang XY, Li BJ (2016). Costunolide induces apoptosis through generation of ROS and activation of P53 in human esophageal cancer Eca-109 cells. J Biochem Mol Toxicol.

[29] Chen JS, Chen BS, Zou ZH, Li W, Zhang YM, Xie JL, Liu CX (2017). Costunolide enhances doxorubicin-induced apoptosis in prostate cancer cells via activated mitogen-activated protein kinases and generation of reactive oxygen species. Oncotarget.

[30] Kim DY, Choi BY (2019). Costunolide-a bioactive sesquiterpene lactone with diverse therapeutic potential. Int J Mol Sci.

[31] Chen L, Bi SN, Hou JZ, Zhao ZJ, Wang CJ, Xie SQ (2019). Targeting p21-activated kinase 1 inhibits growth and metastasis via Raf1/MEK1/ERK signaling in esophageal squamous cell carcinoma cells. Cell Commun Signal.

[32] Whipple RA, Vitolo MI, Boggs AE, Charpentier MS, Thompson K, Martin SS (2013). Parthenolide and costunolide reduce microtentacles and tumor cell attachment by selectively targeting detyrosinated tubulin independent from NF-kappaB inhibition. Breast Cancer Res.

[33] Liu YC, Feng N, Li WW, Tu PF, Chen JP, Han JY, Zeng KW (2020). Costunolide plays an anti-neuroinflammation role in lipopolysaccharide-induced BV2 microglial activation by targeting cyclin-dependent kinase 2. Molecules.

[34] Park E, Song JH, Kim MS, Park SH, Kim TS (2016). Costunolide, a sesquiterpene lactone, inhibits the differentiation of pro-inflammatory CD4(+) T cells through the modulation of mitogen-activated protein kinases. Int Immunopharmacol.

[35] Dong GZ, Shim AR, Hyeon JS, Lee HJ, Ryu JH (2015). Inhibition of Wnt/beta-catenin pathway by dehydrocostus lactone and costunolide in colon cancer cells. Phytother Res.

[36] Ge MX, Liu HT, Zhang N, Niu WX, Lu ZN, Bao YY, Huang R, Yu DK, Shao RG, He HW (2020). Costunolide represses hepatic fibrosis through WW domain-containing protein 2-mediated Notch3 degradation. Br J Pharmacol.

[37] Song MQ, Bode AM, Dong ZG, Lee MH (2019). AKT as a therapeutic target for cancer. Cancer Res.

[38] Itoh N, Semba S, Ito M, Takeda H, Kawata S, Yamakawa M (2002). Phosphorylation of Akt/PKB is required for suppression of cancer cell apoptosis and tumor progression in human colorectal carcinoma. Cancer.

[39] Jin X, Wang C, Wang L (2019). Costunolide inhibits osteosarcoma growth and metastasis via suppressing STAT3 signal pathway. Biomed Pharmacother.

[40] Hu M, Liu L, Yao W (2018). Activation of p53 by costunolide blocks glutaminolysis and inhibits proliferation in human colorectal cancer cells. Gene.

[41] Hua PY, Zhang GX, Zhang YF, Sun M, Cui RJ, Li X, Li BJ, Zhang XY (2016). Costunolide induces G1/S phase arrest and activates mitochondrial-mediated apoptotic pathways in SK-MES 1 human lung squamous carcinoma cells. Oncol Lett.

[42] Bond GL, Hu WW, Bond EE, Robins H, Lutzker SG, Arva NC, Bargonetti J, Bartel F, Taubert H, Wuerl P (2004). A single nucleotide polymorphism in the MDM2 promoter attenuates the p53 tumor suppressor pathway and accelerates tumor formation in humans. Cell.

[43] Mendrysa SM, O'Leary KA, McElwee MK, Michalowski J, Eisenman RN, Powell DA, Perry ME (2006). Tumor suppression and normal aging in mice with constitutively high p53 activity. Genes Dev.

[44] Wang P, Lushnikova T, Odvody J, Greiner TC, Jones SN, Eischen CM (2008). Elevated Mdm2 expression induces chromosomal instability and confers a survival and growth advantage to B cells. Oncogene.

[45] Wade M, Li YC, Wahl GM (2013). MDM2, MDMX and p53 in oncogenesis and cancer therapy. Nat Rev Cancer.

[46] Maya R, Balass M, Kim ST, Shkedy D, Leal JF, Shifman O, Moas M, Buschmann T, Ronai Z, Shiloh Y (2001). ATM-dependent phosphorylation of Mdm2 on serine 395: role in p53 activation by DNA damage. Genes Dev.

[47] Zhang HB, Somasundaram K, Peng Y, Tian H, Zhang HX, Bi DK, Weber BL, El-Deiry WS (1998). BRCA1 physically associates with p53 and stimulates its transcriptional activity. Oncogene.

[48] Liao WL, Lin JY, Shieh JC, Yeh HF, Hsieh YH, Cheng YC, Lee HJ, Shen CY, Cheng CW (2020). Induction of G2/M phase arrest by diosgenin via activation of Chk1 kinase and Cdc25C regulatory pathways to promote apoptosis in human breast cancer cells. Int J Mol Sci.

[49] Giono LE, Resnick-Silverman L, Carvajal LA, St Clair S, Manfredi JJ (2017). Mdm2 promotes Cdc25C protein degradation and delays cell cycle progression through the G2/M phase. Oncogene.

[50] Phesse TJ, Myant KB, Cole AM, Ridgway RA, Pearson H, Muncan V, van den Brink GR, Vousden KH, Sears R, Vassilev LT (2014). Endogenous c-Myc is essential for p53-induced apoptosis in response to DNA damage in vivo. Cell Death Differ.

[51] Rogulski K, Li YJ, Rothermund K, Pu LX, Watkins S, Yi FH, Prochownik EV (2005). Onzin, a c-Myc-repressed target, promotes survival and transformation by modulating the Akt-Mdm2-p53 pathway. Oncogene.

[52] Han CT, Schoene NW, Lei KY (2009). Influence of zinc deficiency on Akt-Mdm2-p53 and Akt-p21 signaling axes in normal and malignant human prostate cells. Am J Phys Cell Phys.

[53] Chou TC, Zhang X, Zhong ZY, Li Y, Feng L, Eng S, Myles DR, Johnson R, Wu N, Yin YI (2008). Therapeutic effect against human xenograft tumors in nude mice by the third generation microtubule stabilizing epothilones. Proc Natl Acad Sci U S A.

[54] Morton JJ, Bird G, Refaeli Y, Jimeno A (2016). Humanized mouse xenograft models: narrowing the tumor-microenvironment gap. Cancer Res.

[55] Wang SM, Sun W, Zhao YJ, McEachern D, Meaux I, Barriere C, Stuckey JA, Meagher JL, Bai LC, Liu L (2014). SAR405838: an optimized inhibitor of MDM2-p53 interaction that induces complete and durable tumor regression. Cancer Res.

[56] Lu JF, McEachern D, Li SQ, Ellis MJ, Wang SM (2016). Reactivation of p53 by MDM2 inhibitor MI-77301 for the treatment of endocrine-resistant breast cancer. Mol Cancer Ther.

[57] Liu S, Tackmann NR, Yang J, Zhang Y (2017). Disruption of the RP-MDM2-p53 pathway accelerates APC loss-induced colorectal tumorigenesis. Oncogene.

[58] Surh YJ (2003). Cancer chemoprevention with dietary phytochemicals. Nat Rev Cancer.

[59] NR C. Guide for the care and use of laboratory animals: National Academies Press; 2011. PMID: 21595115, Bookshelf ID: NBK54050, 10.17226/12910. 21595115

