# Supplementary material for: Targeting AKT with costunolide suppresses the growth of colorectal cancer cells and induces apoptosis in vitro and in vivo
Source: J Exp Clin Cancer Res. 2021 Mar 30;40:114. doi: 10.1186/s13046-021-01895-w (PMC8010944; doi:10.1186/s13046-021-01895-w)
Supplement: Supplementary file 1 — Additional file 1: Figure S1. Analysis of cell proliferation, cell cycle, and apoptosis after treatment with CTD. (a) MTT assay was performed to measure the effect on the cytotoxicity of healthy colon cells named CCD-18Co at 24, 48, 72, and 96 h, respectively. Data are shown as mean ± SD of values from triplicate samples. (*p < 0.05, **p < 0.01, ***p < 0.01) indicate a significant difference compared to control. (b) Corresponding images of anchorage-independent cell growth (magnification, 100×). (c) Representative photographs of the cell cycle after CTD treatment in CRC cells. Cell cycle was measured by flow cytometry in HCT-15, HCT-116, and DLD1 cells after treatment with various costunolide concentrations (0, 2.5, 5, or 10 μM) (d) Representative photographs of apoptosis after CTD treatment in CRC cells. Apoptosis was detected by flow cytometry in HCT-15, HCT-116, and DLD1 cells after treatment with different dose of Costunolide (0, 2.5, 5, or 10 μM). [file 13046_2021_1895_MOESM1_ESM.docx]

**
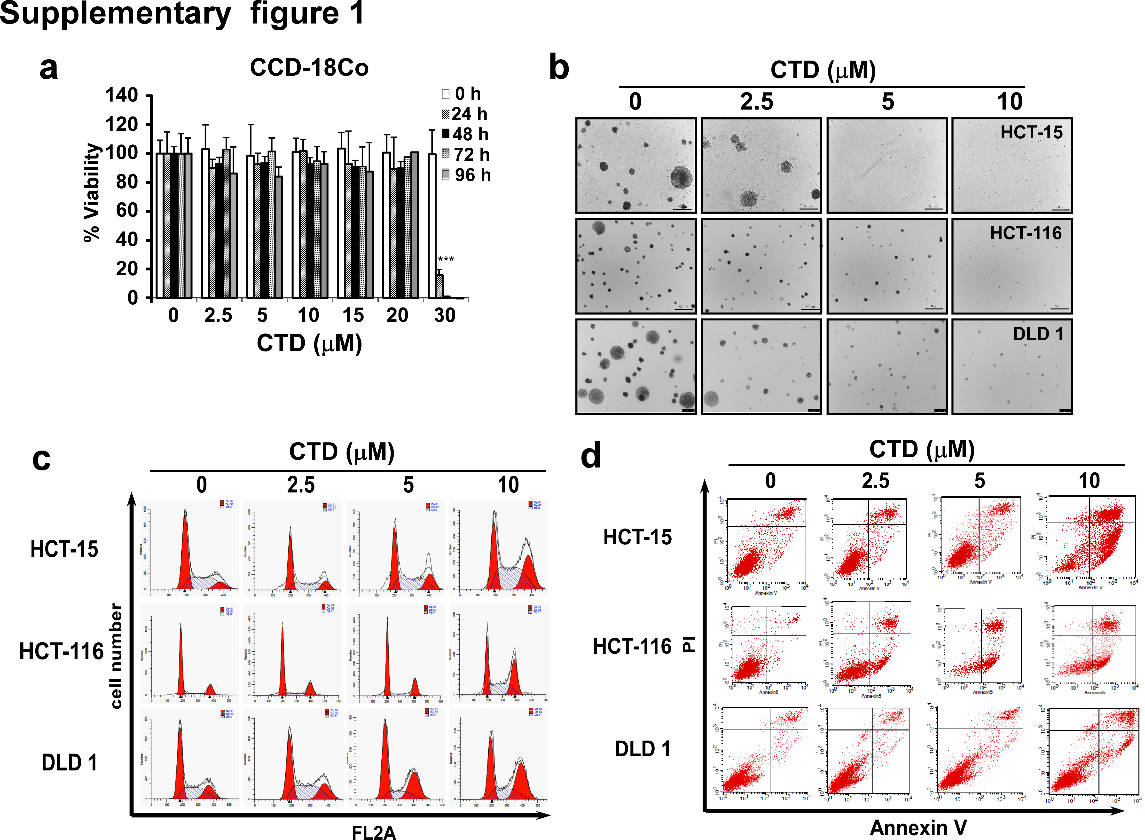
**

**Supplementary Figure 1. Analysis of cell proliferation, cell cycle and apoptosis after treatment with CTD.** **(a)** The MTT assay was performed to measure the effect of CTD on the cytotoxicity of healthy colon cell named CCD-18Co at 24, 48, 72, and 96 h, respectively. Data are shown as mean ± SD of values from triplicate samples. (**p* < 0.05, ***p* < 0.01, ****p* < 0.01) indicate a significant difference compared to control. **(b)** Corresponding images of anchorage‐independent cell growth (magnification, 100×). **(c)** Representative photographs of the cell cycle after costunolide treatment in CRC cells. Cell cycle was measured by flow cytometry in HCT-15, HCT-116, and DLD1 cells after treatment with various costunolide concentrations (0, 2.5, 5, or 10 μM). **(d)** Representative photographs of apoptosis after costunolide treatment in CRC cells. Apoptosis was detected by flow cytometry in HCT-15, HCT-116, and DLD1 cells after treatment with different dose of Costunolide (0, 2.5, 5, or 10 μM).
