# Supplementary material for: Targeting AKT with costunolide suppresses the growth of colorectal cancer cells and induces apoptosis in vitro and in vivo
Source: J Exp Clin Cancer Res. 2021 Mar 30;40:114. doi: 10.1186/s13046-021-01895-w (PMC8010944; doi:10.1186/s13046-021-01895-w)
Supplement: Supplementary file 2 — Additional file 2: Figure S2. CTD suppressed the migration of colon cancer cells by a transmembrane assay. (a) Representative images of the in vitro migration assays in the transwell system. (b) Representative images of the in vitro invasion assay in the transwell system. [file 13046_2021_1895_MOESM2_ESM.docx]

**
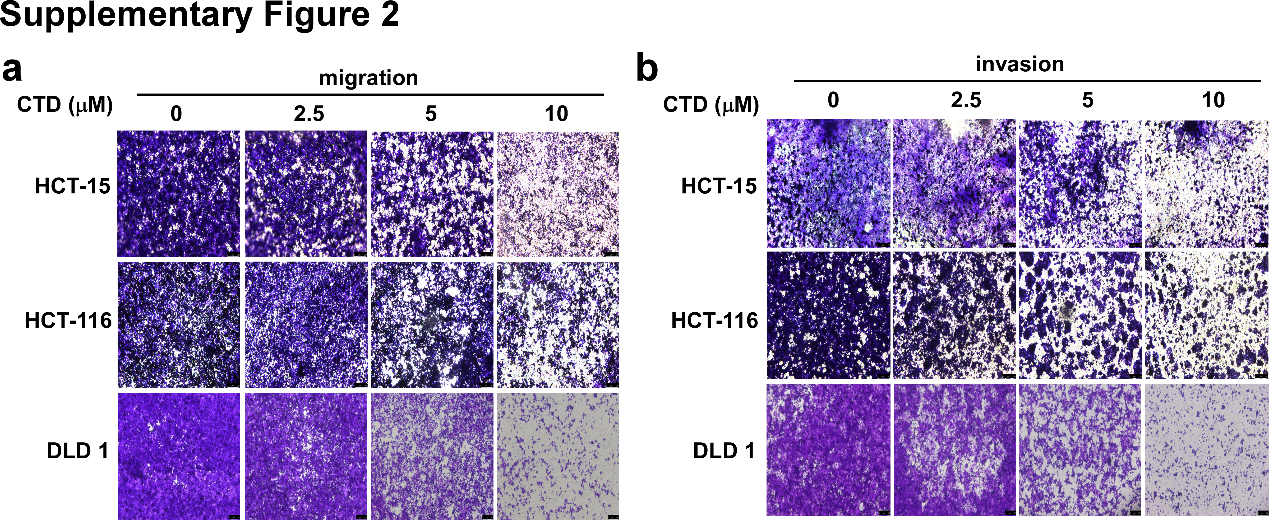
**

**Supplementary Figure 2. CTD suppressed the migration of colon cancer cells by a transmembrane assay.** **(a)** Representative images of the *in vitro* migration assays in the transwell system. **(b)** Representative images of the *in vitro* invasion assay in the transwell system.
