# Supplementary material for: Targeting AKT with costunolide suppresses the growth of colorectal cancer cells and induces apoptosis in vitro and in vivo
Source: J Exp Clin Cancer Res. 2021 Mar 30;40:114. doi: 10.1186/s13046-021-01895-w (PMC8010944; doi:10.1186/s13046-021-01895-w)
Supplement: Supplementary file 3 — Additional file 3: Figure S3. AKT is frequently overexpressed in CRC. (a). The expression of phosphorylated AKT (Ser473) and total AKT (pan) was examined by IHC analysis using a CRC tumor microarray (100× magnification). The top panels show the quantitation of all samples. (b). Representative images of IHC staining on a CRC tumor microarray are shown. The AKT and p-AKT expression are stained in brown, and the nuclei are stained blue with hematoxylin. Two images are shown from each group (magnification, 100×). (c). The mRNA level of of AKT1, AKT2, and AKT3 expression was calculated from the data set GSE21815 (N = 123 pairs, which were examined using cDNA microarray from primary CRC and paired normal tissues). (*p < 0.05, **p < 0.01, ***p < 0.001) indicate a significant difference compared to control. (d). The overall survival time of patients with high or low AKT expression (http://gepia.cancer-pku.cn/). (e). Western blot analysis in one normal colon cell line (CCD-18 Co) and other normal skin (HaCaT) and six other types of cancer cell lines (colon cancer, lung cancer, breast cancer, liver cancer, prostate cancer, and melanoma). [file 13046_2021_1895_MOESM3_ESM.docx]

**
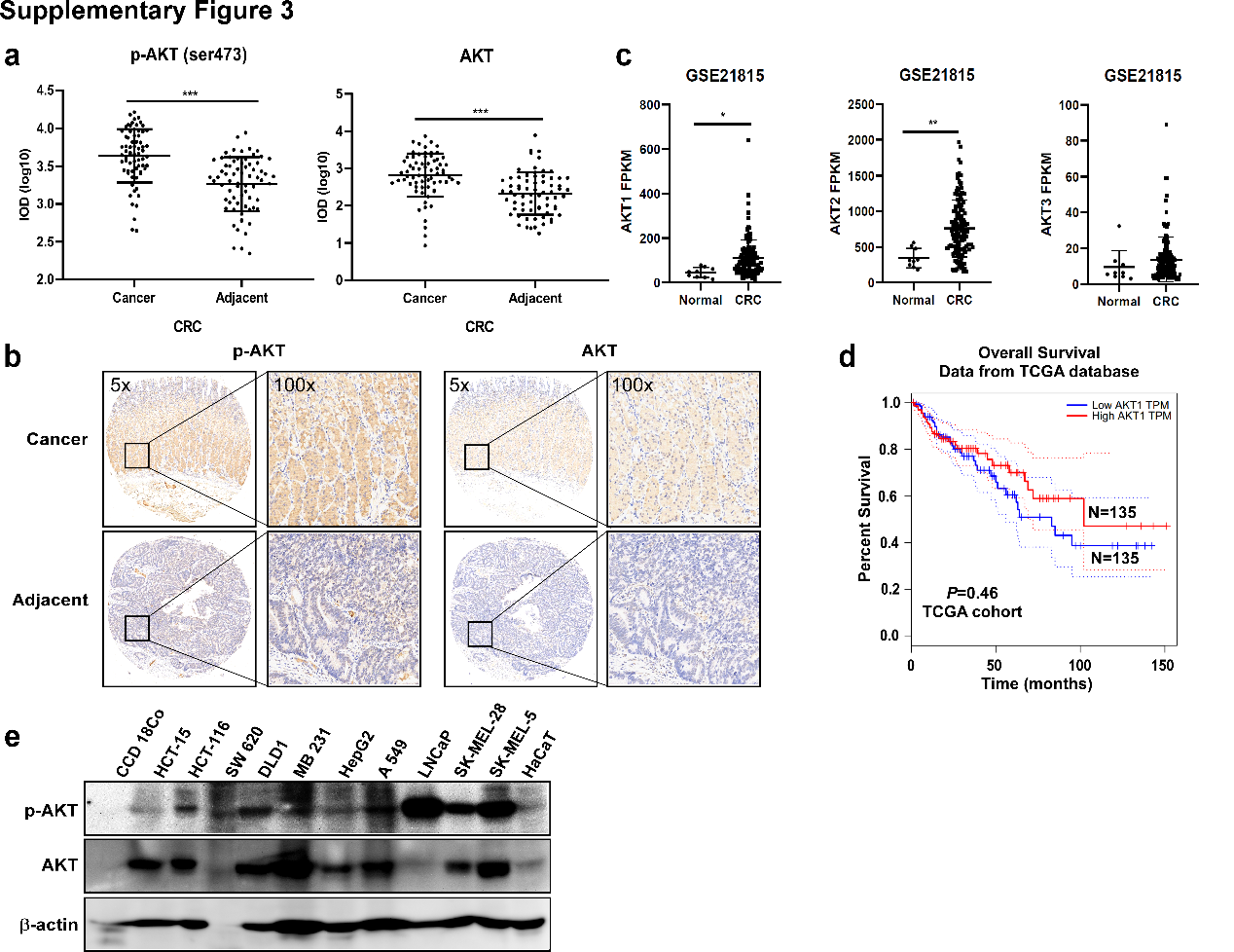
**

**Supplementary Figure 3. AKT is frequently overexpressed in CRC. (a).** The expression of phosphorylated AKT (Ser473) and total AKT (pan) was examined by IHC analysis using a CRC tumor microarray (100×magnification). The top panels show the quantitation of all samples. **(b)**. Representative images of IHC staining on a CRC tumor microarray are shown. The AKT and pAKT expressions are stained in brown, and the nuclei are stained blue with hematoxylin. Two images are shown from each group (magnification, 100×). **(c).** The mRNA level of of AKT1, AKT2, and AKT3 expression was calculated from the data set GSE21815 (N = 123 pairs, which were examined using cDNA microarray from primary CRC and paired normal tissues). (**p* < 0.05, ***p* < 0.01, ****p* < 0.001) indicate a significant difference compared to control. (**d).** The overall survival time of patients with high or low AKT expression (<http://gepia.cancer-pku.cn/>). **(e).** Western blot analysis in one normal colon cell line (CCD-18 Co) and other normal skin (HaCaT) and six other types of cancer cell lines (colon cancer, lung cancer, breast cancer, liver cancer, prostate cancer, and melanoma).
