# Supplementary material for: Targeting AKT with costunolide suppresses the growth of colorectal cancer cells and induces apoptosis in vitro and in vivo
Source: J Exp Clin Cancer Res. 2021 Mar 30;40:114. doi: 10.1186/s13046-021-01895-w (PMC8010944; doi:10.1186/s13046-021-01895-w)
Supplement: Supplementary file 4 — Additional file 4: Figure S4. Confirm the AKT is the target of CTD in knockdown cells. (a). Representative colony pictures after AKT1/2 knockdown with or without CTD treatment. (b). Representative images of in vitro migration and invasion assays in the transwell system after the knockdown of AKT1/2. [file 13046_2021_1895_MOESM4_ESM.docx]

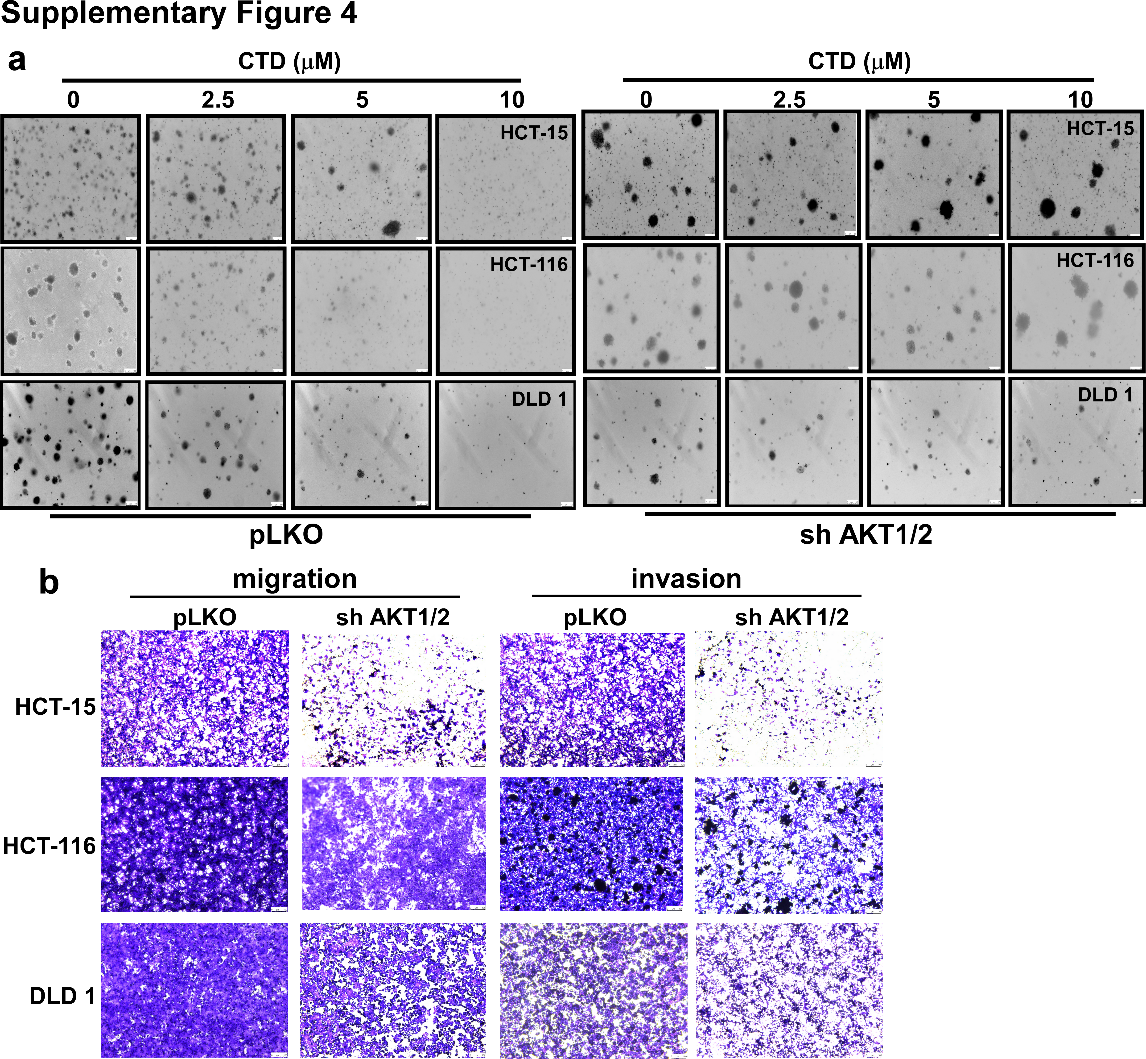


**Supplementary Figure 4. Confirm the AKT is the target of CTD in knockdown cells.** **(a).** Representative colony pictures after AKT1/2 knockdown with or without costunolide treatment. **(b).** Representative images of *in vitro* migration and invasion assays in the transwell system after the knockdown of AKT1/2.
