# Supplementary material for: Targeting AKT with costunolide suppresses the growth of colorectal cancer cells and induces apoptosis in vitro and in vivo
Source: J Exp Clin Cancer Res. 2021 Mar 30;40:114. doi: 10.1186/s13046-021-01895-w (PMC8010944; doi:10.1186/s13046-021-01895-w)
Supplement: Supplementary file 5 — Additional file 5: Figure S5. Effects of AKT1/2 knockdown on the intranuclear content of p53 and MDM2. The cells were stained for p53 (red) and MDM2 (Green), and the nuclei were counterstained with DAPI (blue). Localization of p53 and MDM2 expression in the cells are shown in the merged image. Scale bar = 100 μm. [file 13046_2021_1895_MOESM5_ESM.docx]

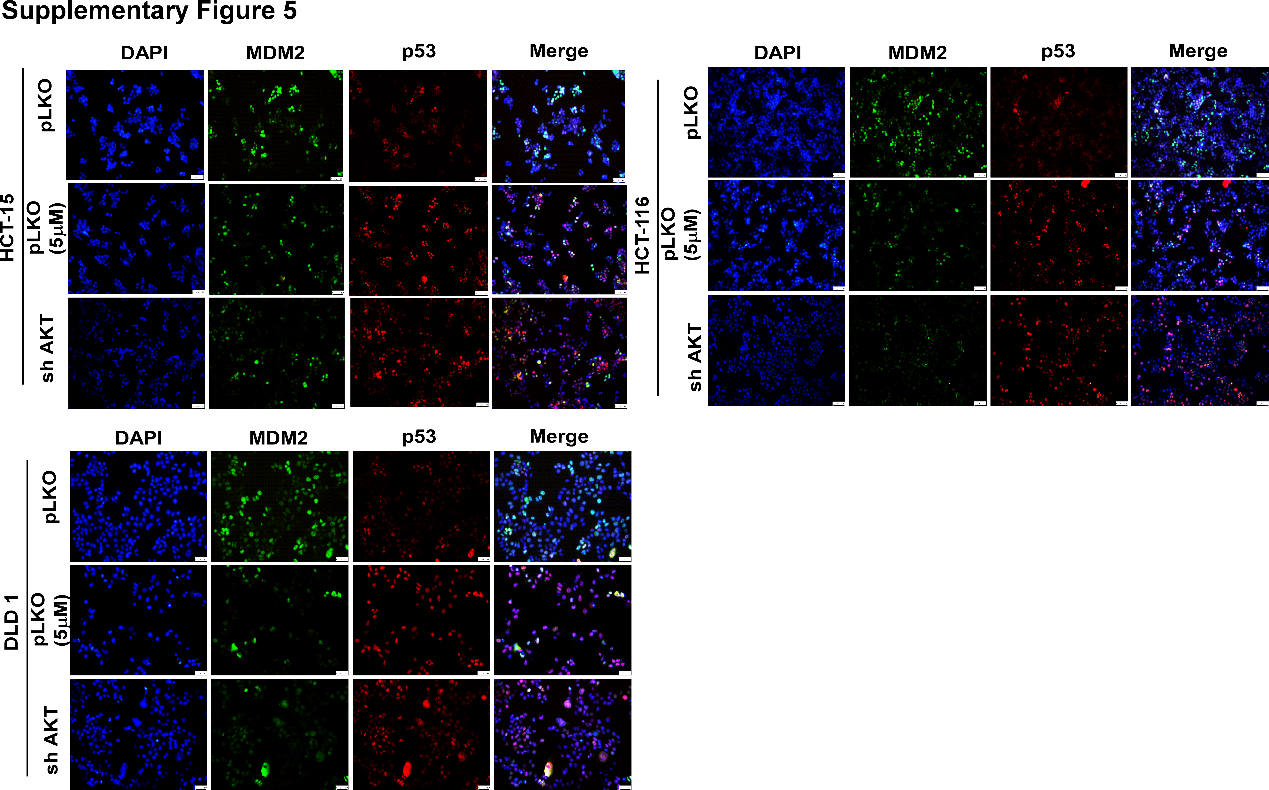


**Supplementary Figure 5. Effects of AKT1/2 knockdown on the intranuclear content of p53 and MDM2.** The cells were stained for p53 (red) and MDM2 (Green), and the nuclei were counterstained with DAPI (blue). Localization of p53 and MDM2 expression in the cells are shown in the merged image. Scale bar = 100 μm.
