# Supplementary material for: Targeting AKT with costunolide suppresses the growth of colorectal cancer cells and induces apoptosis in vitro and in vivo
Source: J Exp Clin Cancer Res. 2021 Mar 30;40:114. doi: 10.1186/s13046-021-01895-w (PMC8010944; doi:10.1186/s13046-021-01895-w)
Supplement: Supplementary file 6 — Additional file 6: Figure S6. AKT is a therapeutic target of CRC cells. (a). Overexpression of AKT1/2 was detected by Western blot in CRC cell lines. (b). The effect of proliferation by MTT assay at different time points in CRC cell lines after overexpression of AKT1/2. (c). Anchorage-independent cell formation was assessed after treatment with different doses of CTD in cells expressing mock or AKT1/2. (d). Representative colony images after AKT1/2 over-expression with CTD treatment. (e). The quantification showing the effect of migration by transmembrane assay after overexpression of AKT1/2. (f). The quantification showing the effect of invasion by the transmembrane assay after overexpression of AKT1/2. (g). Representative images of in vitro migration and invasion assays in the transwell system. Data are shown as mean ± SD of values from triplicate samples. (*p < 0.05, **p < 0.01, ***p < 0.001) indicate significant differences compared to control. [file 13046_2021_1895_MOESM6_ESM.docx]

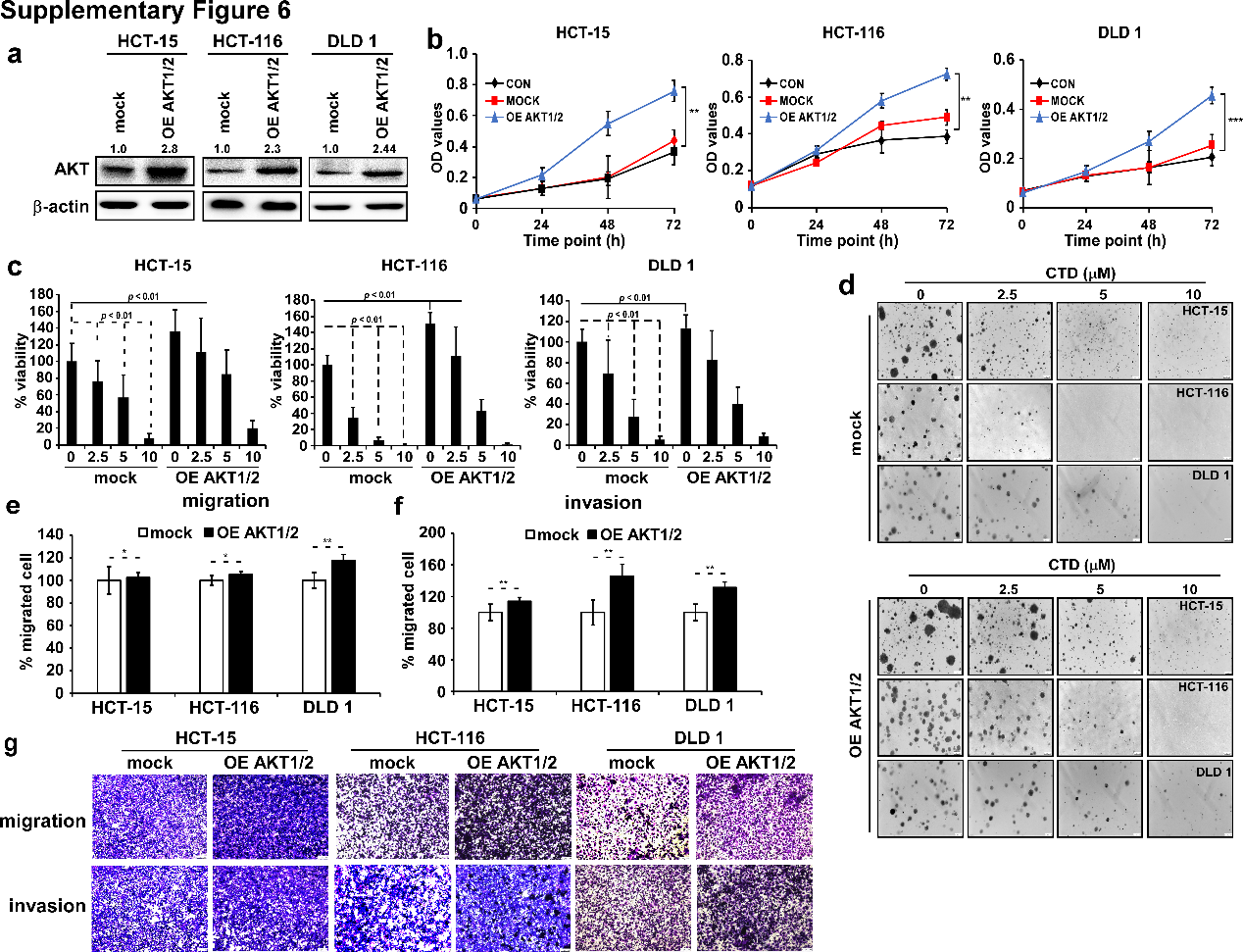


**Supplementary Figure 6. AKT is a therapeutic target of CRC cells. (a).** Overexpression of AKT1/2 was detected by Western blot in CRC cell lines. **(b).** The effect of proliferation by MTT assay at different time points in CRC cell lines after overexpression of AKT1/2. **(c).** Anchorage-independent cell formation was assessed after treatment with different doses of CTD in cells expressing mock or AKT1/2. **(d).** Representative colony images after AKT1/2 over-expression with Costunolide treatment. **(e).** The quantification showing the effect of migration by transmembrane assay after overexpression of AKT1/2. **(f).** The quantification was showing the effect of invasion by the transmembrane assay after AKT1/2. **(g).** Representative images of *in vitro* migration and invasion assays in the transwell system. Data are shown as mean ± SD of values from triplicate samples. (**p* < 0.05, ***p* < 0.01, and ****p* < 0.001) indicate significant differences compared to control.
